# ConfocalCheck - A Software Tool for the Automated Monitoring of Confocal Microscope Performance 

**DOI:** 10.1371/journal.pone.0079879

**Published:** 2013-11-05

**Authors:** Keng Imm Hng, Dirk Dormann

**Affiliations:** MRC Clinical Sciences Centre, Faculty of Medicine, Imperial College London, London, United Kingdom

## Abstract

Laser scanning confocal microscopy has become an invaluable tool in biomedical research but regular quality testing is vital to maintain the system’s performance for diagnostic and research purposes.

Although many methods have been devised over the years to characterise specific aspects of a confocal microscope like measuring the optical point spread function or the field illumination, only very few analysis tools are available. Our aim was to develop a comprehensive quality assurance framework ranging from image acquisition to automated analysis and documentation. We created standardised test data to assess the performance of the lasers, the objective lenses and other key components required for optimum confocal operation.

The ConfocalCheck software presented here analyses the data fully automatically. It creates numerous visual outputs indicating potential issues requiring further investigation. By storing results in a web browser compatible file format the software greatly simplifies record keeping allowing the operator to quickly compare old and new data and to spot developing trends.

We demonstrate that the systematic monitoring of confocal performance is essential in a core facility environment and how the quantitative measurements obtained can be used for the detailed characterisation of system components as well as for comparisons across multiple instruments.

## Introduction

Since the release of the first commercially available instruments more than 25 years ago laser scanning confocal microscopy has rapidly become a standard tool in many areas of research in the life sciences and medicine [1,2]. The combination of a diffraction limited spot of laser light - scanning across a fluorescently labelled sample - with the confocal pinhole – an aperture in a conjugate image plane placed in front of the light detector – allows the effective rejection of out-of-focus fluorescence light emanating from excited fluorophores outside the focal volume [3,4]. From these blur-free ‘optical sections’ the three-dimensional structure of both fixed and living biological samples can be rapidly reconstructed. This has been invaluable in countless studies and to date more than 45600 articles have been published on the subject [5].

Confocal microscopes are complex instruments consisting of many optical, mechanical and electronic components that all need to be properly aligned and calibrated before accurate data can be recorded [4]. Despite their popularity and wide spread use there are very few if any tools provided by the manufacturers themselves to monitor confocal performance following the purchase of a new system. It might just be a slide with a fluorescently labelled section of plant tissue (Convallaria spec., ‘lily of the valley’) which provides ‘pretty’ multi-colour images but the use of these biological samples as test specimens for the evaluation of a confocal microscope is very limited [6,7]. 

While imaging experts and staff of centralised microscopy facilities would be able to recognise many of the issues adversely affecting confocal image acquisition there are far more confocal instruments in individual research labs where this specialised support might not be readily available. 

How can the average user, the cell or developmental biologist be confident that the images acquired on a confocal microscope are actually reflecting the biology of the sample rather than the variability of the instrument? Due to the increasing demand for quantitative analysis of biological samples and the complexity of biological research with experiments stretching over many months or years or even spread over several laboratories it is essential to ensure the accuracy and comparability of the data recorded on a confocal microscope [6,8]. When performing quantitative fluorescence imaging the main source of concern would be temporal and spatial signal intensity variations introduced by the instrument itself (which are by no means specific to the confocal microscope) [8,9,10]. Even the probably most common and basic confocal application, studying the spatial distribution or colocalisation of two different fluorescently labelled antibodies could easily be invalidated if the system was misaligned or the wrong objective lens used. 

For the systematic analysis of confocal performance a wide range of tests have been developed, mostly relying on inexpensive samples and yet providing a wealth of information on the state of the instrument and individual components [7,8,11,12,13,14,15,16,17,18,19,20,21,22,23,24,25,26,27,28,29]. Although there are many differences in the design of the commercially available confocal microscopes - and each of them available with a multitude of possible configurations - the following parameters are commonly checked for quality control purposes: 

Lasers: Measuring the laser power directly on the microscope stage with a power meter is used to assess not only the status of the lasers themselves but probes the alignment of entire excitation light path with all the intermediate optical elements and light guides [20,21]. Short term laser stability and noise can be measured with time lapse recordings (in fluorescence, reflection or transmitted light mode)[19,24]. The observed intensity fluctuations should ideally be negligible compared to the signal variations that need to be measured in the biological sample.

Field illumination: The field pattern is observed by illuminating a homogeneously fluorescent sample, the intensity distribution should be relatively uniform with the maximum signal in the centre but this will depend of the objective lens, alignment of the lasers, any corrective optics for UV/405nm lasers etc [7,13]. For quantitative measurements any unevenness in the field should be avoided or corrected for.

Image distortion: A grid pattern of small squares is used to test the XY scanning mechanism that moves the laser beam across the sample [13,19]. Any deviation from the square shape would indicate a distortion in the image that would need to be fixed.

Spectral registration: This is tested by imaging multi-colour fluorescent beads in three dimensions [7]. For colocalisation experiments the bead images in the different colour channels should coincide with very little deviation. Although dependent on the type of objective lens used spectral registration will be affected by the alignment of other components like the lasers as well.

Point spread function (PSF): The PSF is obtained by recording sub-resolution fluorescent beads in three dimensions. It is used to determine the axial and lateral resolution of the microscope and to identify problems in the imaging system in particular the objective lenses [27,30].

Axial resolution: The axial resolution is used to assess the optical sectioning capability of the instrument and can be obtained from imaging a thin fluorescent film [26,28,31] or the reflection of laser light from a mirror [7,13]. Axial resolution has been described as a ‘gold standard’ and is used by some manufacturers to check whether the system is working according to specifications [7]. By comparing the reflection bands of multiple laser lines the axial chromatic correction of the objective lenses can be revealed - how well light of different wavelengths is focused in the same plane, important again for colocalisation experiments [13,20].

There are many excellent guidelines and protocols included in the literature cited above on how to acquire suitable data sets with the test samples and how to interpret the results. Some parameters like resolution can easily be measured using the basic quantification tools available in the confocal software packages but this can be tedious, subjective and time consuming. And yet there are very few software tools for the automated quantitative analysis of the performance data. Some programs have been developed only for a specialised purpose, for example to analyse the PSF [32], or to provide the detailed quantification of the Z-sectioning capability using the SIPchart methodology [26,31,33]. To date only the MetroloJ plugin for ImageJ can process a wider range of data, measuring the PSF, bead colocalisation, field illumination and axial resolution [34,35].

As none of these tools were able to capture the range of issues we had encountered on the confocal instruments in our microscopy facility over the years we decided to develop the ConfocalCheck software which integrates many previously proposed analysis methods into a single easy to use package. We focussed on three general topics - the lasers, the objective lenses and other important mechanical components present on a modern confocal microscope. This comprised the scanning galvos, the Z-focus drives, the motorised microscope stages and the spectral detector units that are specific to Leica SP confocal microscopes. The interplay of all these components is essential for the optimum operation of the microscope and can easily be monitored on a regular basis. 

The protocols for the acquisition of the test data were standardised to provide the basis for the fully automated and quantitative analysis with the ConfocalCheck software, without user intervention and bias. A visual output for most parameters assists in the quick detection of potential problems while additional files in the HTML format are created for record keeping and long-term monitoring. This should be of particular benefit to core facility staff involved in the maintenance of multiple confocal units. Even during the initial testing phase on the six single-point scanning confocal systems available in our unit we discovered many issues that required further investigation by service engineers suggesting that there are many underperforming systems out there. This can be a serious problem especially when there is no trained staff carrying out regular system checks. Unless the problem is so severe that it is noted by the average user many faults could remain undetected for some time.

We describe the acquisition and analysis of the confocal test data sets, illustrated with examples from the microscopes available in our facility. Regular and systematic checks using the tools described in this report should assist even the inexperienced user in identifying potential problems that might need to be addressed. Furthermore, the quantitative measurements obtained with ConfocalCheck give the operator the means to characterise and compare individual components across multiple instruments.

## Materials and Methods

### Sources of slides and materials

A slide with a reflective square grid pattern (9.9µm x 9.9µm) was taken from a set of Deltavision calibration slides (Applied Precision, Inc.; similar grid slides: planotec silicon test specimen S1934, Agar Scientific). We used the red fluorescent slide of that kit (Excitation 590nm, Emission 650nm) for the field illumination tests of the 543/561/594/633nm laser lines and the blue and green fluorescent Chroma slides (part no. 92001, Chroma technology Corp.) or the equivalent Deltavision slides for testing the 405nm and 488nm field illumination [7,20]. Glass slides (76x26mm) and No 1.5 coverslips (22x22mm, Menzel-Gläser) were obtained from Fisher Scientific. As most modern objective lenses are designed to be used with 170µm coverslips high-precision cover glasses could also be considered for best performance (No. 1.5H, thickness 170µm±5µm; Marienfeld Superior cat.no. 0107052 or Zeiss cat.no. 474030-9020-000). 

Front surface mirrors were from Edmund Optics Ltd (part no: 32368, 22x22mm), 175nm green PS-speck beads (P-7220), 1µm Tetraspeck beads (T-7282) and Prolong Gold mounting medium (P36934) from Life Technologies Corporation. 

Laser power was measured with a Fieldmaster power meter (part no. 33-0506-000, Coherent Inc.) where the LM-2 VIS sensor (part no. 1098298, wavelength range: 405-1070nm) had been mounted on a custom made slide-sized holder to simplify power measurements directly on the microscope stage (see 21 for possible designs). 

Stage temperature was monitored with a ‘USB-Temp’ PC Thermometer, (31.1026, TFA Dostmann GmbH, Germany), room temperature with a RMS300 Weatherstation (Oregon Scientific).

### Preparation of Slides

The mirror slide was prepared by gluing the mirror directly onto a glass slide [7]. 175nm PS-speck beads (green only) and 1µm Tetraspeck bead stock tubes were vortexed, diluted 1:10000 in distilled water and vortexed again. 5µl of each suspension was placed in the centre of the same coverslip and allowed to dry [21,32]. The coverslip was carefully lowered on a slide with a 5µl drop of Prolong gold mounting medium which was allowed to cure for 24h at room temperature in darkness. All fluorescent plastic slides and the mirror slide were covered with no 1.5 coverslips held in place with a small drop of immersion oil (n=1.518, Zeiss Immersol 518F) as described [7]. For inexperienced users it might be useful to prepare additional slides with more beads (1:100 dilution) as finding the PS-speck beads can be challenging.

### Performance checks - data acquisition

The following confocal systems were available in our microscopy facility and used for evaluation: Leica (Leica Microsystems) SP1 and SP2 with dichroic beamsplitters, 2× Leica SP5 with AOBS (acousto optical beam splitter), Leica SP5 II with AOBS. Additonal tests were carried out on a Zeiss LSM510 Meta system (Carl Zeiss Microscopy, Jena, Germany), a Zeiss LSM780 and a Nikon A1R (Nikon Instruments) microscope. On another Deltavision Core (Applied Precision) deconvolution microscope we only measured the motorised stage performance. 

The confocal systems including all the lasers were switched on at least 1h prior to testing and all objective lenses were first inspected and cleaned with absolute ethanol as required.

On the Leica systems a single experiment file (.lif/.lei) should be created for each objective lens. For the Zeiss (.lsm/.czi), Nikon (.nd2) and TIFF (.tif) files separate folders should be created for each objective lens with each folder containing the relevant image files eg. “bead.lsm”, “psf.nd2”, “bead.tif”. Importantly, the file and folder names must not contain any spaces as that causes problems with data import in ImageJ/Fiji. 

A detailed step-by-step protocol describing the image acquisition is available in Protocol S1 as the automatic analysis with the ConfocalCheck macro is dependent on suitable input data. The general procedures are described below.

#### Lasers – Laser power

The power measurements were taken in the specimen plane on the microscope stage using a low magnification 10× objective [20,21] with laser beam blanking disabled. Having direct meter readings was very useful when communicating with service engineers to assess whether a callout is required. Alternatively one could for example measure the reflection of the different laser lines from the mirror slide. By always using the same fixed scan parameters and fixed settings for the detector gain/offset and the laser power changes in image intensity should reflect changes in laser intensity. At fixed laser power settings one could also note the detector gain required to achieve saturation, this could be done with the mirror slide, with a fluorescent sample or even without a slide using the transmitted light detector (see section below).

#### Lasers – Laser stability

Laser stability was measured from time lapse recordings for all lasers using the transmitted light PMT (photomultiplier tube) and the 10× lens [8,19]. First the transmitted light path was properly adjusted according to Köhler. PMT gain and offset were kept the same for all laser lines, with the PMT gain as low as possible to keep detector noise low. Image brightness for each wavelength was adjusted with the AOTF (acousto-optical tunable filter) to an intermediate grey intensity to monitor intensity fluctuations during the time course. Using the sequential scan mode scanning only with one laser line at a time images were recorded every 20s for 2 hours at 256×256 pixels and at the highest zoom settings to avoid intensity variations within the images due to uneven field illumination. Long term laser stability was measured overnight or as required. The image series was renamed *laser* for the subsequent automatic analysis.

#### Objective lens – axial resolution and chromatic correction

Axial resolution and axial chromatic correction was measured for all objective lenses by using the reflection of laser light from the mirror slide [7,13,21]. To detect the reflected light we used 10-20nm wide detection windows centred on the main laser lines (eg 488/561/633nm). Depending on the number of available detection channels between 3 and 5 lasers lines were recorded simultaneously. After focussing on the mirror surface the acquisition was changed to the XZ line scan mode. The confocal pinhole diameter was set to 1 Airy unit, however when trying to obtain the highest possible Z resolution in reflection mode the confocal pinhole was set to its minimum diameter [7]. The zoom factor was increased (about 10-40× zoom to achieve a z step size of 0.015-0.03µm when using a Leica Z-galvo) and the bright horizontal reflection band placed in the middle of the field using the microscope focus control. XZ images were recorded with the following combinations of laser lines (depending on the system configuration): 

1. 405/488/543(561)nm; 2. 488/543(561)/633nm. As most instruments only had three PMTs available we performed these two scans to avoid using sequential scanning which would have introduced a time delay and possibly changes in the Z position due to vibration or focus drift. On a 4 or 5 channel system all these laser lines can be evaluated in a single scan. The scans starting with the 405nm line were renamed *axial405*, the scans starting with the 488nm line were renamed *axial488*.

#### Objective lens – field illumination

Field illumination was tested for each objective lens and for all the main laser lines (for example 405/488/561/633nm) with the fluorescent plastic slides [8,13,21]. The slides were mounted and the slide surface identified by looking for the brightest fluorescence signal. The actual recordings were taken at an offset focussing 30-75 µm into the slide depending on the objective (10×: 75µm; 20×: 50µm; 40×: 40µm; 63×: 30µm; 100×: 30µm) to reduce the uneven intensity distribution observed on the slide surface [7,8]. The zoom factor was set to 1 to obtain the maximum field size, image size to 512×512 pixel and pinhole size set to 1 Airy unit. Low laser power was used to avoid bleaching and due to high PMT gain images were averaged (4× line/frame average). The recorded images were renamed *field405, field488* etc.

#### Objective lenses – bead colocalisation

Spectral registration was measured using 1µm Tetraspeck beads [8]. Beads on the coverslip (closest to the objective lens) were selected using low intensity epi-fluorescence illumination and centred in the field of view. 

The pinhole diameter was set to 1 Airy unit, image size to 256×256 pixels and XY resolution to about 0.03µm (zoom factor 16-64×)[7]. Laser power was kept reasonably low to avoid bleaching and gain/offset adjusted to optimize the dynamic range. Very noisy images were averaged four times. Using the XYZ scan mode with sequential scanning for 3-4 wavelengths (eg 405/488/561/633nm) we recorded Z stacks with the total Z height and Z step size depending on the objective lens (eg. 10×: 1µm steps; 63× oil: 0.15µm steps). The oversampling in the XY and in the Z direction is required for analysis and visualization purposes. Stacks were renamed *bead*. Only one bead per stack is permitted as the analysis software does not distinguish between multiple beads within the field of view.

#### Objective lenses – point spread function

The point spread function was measured using green 175nm PS-speck beads excited with the 488nm laser line [13,21,27]. Beads closest to the coverslip were selected and centred in the field of view. The other acquisition settings were the same as for the Tetraspeck beads described above. The 3D image stack was renamed *psf*. Only one bead per stack is permitted as the analysis software does not distinguish between different beads.

### Testing other mechanical components

#### Spectrophotometer accuracy

The accuracy of the spectral slider movement in the spectrophotometer unit of the Leica SP systems was measured with the 10× lens and the mirror slide for three laser lines - 488, 543 or 561, 633nm – again in reflection mode [13,19]. After focussing on the mirror surface, the confocal pinhole was fully opened, image size set to 256×256 pixel and zoom to 8×. 

The lambda scan mode (XYλ) was selected and the detection window width set to 5nm. The PMT offset was left at 0. The detection window was moved to the different laser lines and the gain adjusted for each PMT so that the reflection image at each line was below saturation for the most sensitive PMT. AOTF settings for each laser line were adjusted to obtain similar peak intensities.

The lambda scan was performed from 470 to 670nm in 2nm intervals in 100 steps on the SP1/SP2 systems (SP5: 470-668nm, 3nm intervals, 67 steps). This was repeated for all spectral detectors available keeping the same AOTF settings and the same gain/offset values The AOTF/gain/offset settings should be optimised first by testing the reflection images for all the different detectors and laser lines to ensure that there is no saturation while running the lambda scan. The individual scans were renamed *scanpmt1, scanpmt2*, etc.

#### XY scanning galvos

The accuracy of the XY scanning galvos was assessed using the 10x lens with the grid slide [13,19]. Using the 488nm line in reflection mode and with the pinhole diameter set to 1 airy unit we focussed on the grid pattern. If the grid lines were not parallel to the scan field the slide was rotated either manually or using the scan field rotation optics. The zoom was adjusted (8×) so that the width and the height of the squares in the grid pattern could be properly assessed. A single image was recorded with 1024×1024 pixel and renamed *grid*.

#### XY motorised stage

The performance of the motorised microscope stages was assessed by repeatedly recording either different fluorescent beads at different locations of the bead slide or the same bead at different locations within the same field of view. We used the 20×/air objective lens to image 1µm Tetraspeck beads with the 633nm line to reduce photobleaching. Pinhole size was set to 1 Airy unit, image size 256x256 pixel, resolution 0.1µm/pixel. To measure unidirectional stage repeatability over a larger area three different beads were defined (5-10mm apart) and a timelapse recording started that visited these positions 100 times. The recordings of the different positions were named *stage1, stage2* and *stage3* in the Leica .lif/.lei experiment files and the TIFF format and *stage* in the other file formats. 

To measure stage accuracy as well as repeatability over a small movement range we used one bead and shifted its position once or twice within the field of view by a given value (eg. 10µm in X; 10µm in Y). A timelapse recording visited these positions 100 times. The three bead positions were named *stageacc1, stageacc2* and *stageacc3* in the Leica .lif/.lei experiment files and the TIFF format and *stageacc* in the other file formats. These tests could also be performed at higher magnification or on larger samples like multi-well plates to assess performance over long distances.

#### Z-galvo stability

The stability of the Z-galvo available on the Leica SP systems was measured similarly to the axial resolution described above except that only the reflection of the 488nm line was used with a high NA 40×-100× objective [7,19,29]. After focussing on the surface of the mirror slide, the scan mode was switched to XZT and the pinhole diameter set to 1 Airy unit (or to the minimum diameter when trying to achieve maximum Z resolution). The horizontal reflection band was placed in the middle of the image at a Z-resolution of 0.015-0.03µm, image size 256x256 pixels. Time-lapse series with 2s interval were recorded for at least 10min but ideally for one hour or longer. The image series was renamed *zgalvo*. 

Table 1 summarises which tests were performed with different objective lenses.

**Table 1:** Summary of the routine confocal checks performed with different objective lenses.

	**Performance test**									
**Objective lens**	Laser power	Laser stability	Axial resolution and colocalisation	Field illumination	Bead colocalisation	Point spread function (PSF)nbsp;	Lambda scan^(1)^	XY galvos	XY stage	Z galvo stability^(1)^
10×	●	●	●	●	●	●	●	●		
20×			●	●	●	●			●	
40×			●	●	●	●				●*
63×			●	●	●	●				●*
100×			●	●	●	●				●*

* Z galvo stability is only tested with one of the high NA objectives.

^(1)^ These measurements are only applicable to the Leica SP confocal systems SP1/SP2/SP5.

### Design and Implementation of the analysis software

The processing and analysis of the image data was performed automatically with minimal user intervention using the custom-written ConfocalCheck macro (Macro S1). We tested ConfocalCheck with ImageJ (version 1.46r)[35] and Fiji (version 1.47h)[36] on a Windows XP 32bit Dell Optiplex 620 PC with 2GByte RAM and a Windows 7 64bit Dell Optiplex 980 computer. The LOCI bioformats plugin was required for the import of some of the confocal files and to obtain the metadata (release version 4.4.8, built 1May2013) [38]. This bioformats version works fine, however as there are frequent revisions often affecting the name tags used to obtain the metadata values different versions might require simple changes to the macro code. 

Before using ConfocalCheck for the first time some system specific details have to be edited in the configuration file (ConfocalCheck_Configuration.txt; File S1) as detailed in Protocol S2. This only involves providing a list of the installed objective lenses and specifying the lasers used for various performance tests.

A step-by-step guide on running ConfocalCheck, details of the output files and the measurements it creates are provided in Protocol S3. Several datasets are available to evaluate the software (Files S2, S3, and S4).

ConfocalCheck searches for the different keywords eg. *laser*, *bead*, *psf* etc in the experiment files (Leica .lif/.lei) or in the experiment folders with the instrument specific data files (Zeiss .lsm/.czi; Nikon .nd2). Once found appropriate metadata are retrieved and the images loaded and processed. TIFF images or stacks acquired on other systems should be labeled by the appropriate keyword followed by the extension .*tif* for example *bead.tif* or *psf.tif*. 

All measurements are saved in tab delimited text files that can be opened with any text editor or spreadsheet software. 

Additionally HTML files containing the result images and measurements can be created at a user defined location. This is done for each confocal system with a separate HTML file for each objective. The HTML files are amended when a new analysis is carried out at a later date providing a fast and simple way of tracking the different performance parameters over time. 

#### Laser stability

For each laser line the average image intensity and the standard deviation are measured at each time point and plotted versus time [19,21]. The minimum and maximum intensities are determined as well as the maximum percentage change. A number of output images are created: a plot containing the mean intensity versus time, a rescaled version to visualize smaller fluctuations and a plot of the standard deviation versus time as a measure of how laser noise changes over time. To measure laser noise on a very short time scale the pixel intensities along a single horizontal line scan in the first image of the time lapse are recorded and mean, standard deviation and CV (coefficient of variation) values calculated [21]. The intensity profiles are plotted as pixel intensity versus pixel position. 

To quantify laser intensity fluctuations over time scales from µs to hours we calculated the differences between adjacent data points [24] and created histograms of the percentage changes. For the µ-second time scale we obtained the intensity data from horizontal line plots of the first 100 images, the millisecond time scale was derived from 100 vertical intensity plots from the same images. For the other time scales we compared the mean intensities of images recorded 20s, 1min, 10min and 1 hour apart.

#### Axial resolution

For the axial resolution measurements a line is manually drawn perpendicular to the horizontal reflection pattern [7,13]. Alternatively this can be done automatically through the centre of the image. The smoothened intensity profile along this line is derived by averaging over 10 pixels in X direction and the peak Z position and the FWHM (Full Width at Half Maximum) for each laser line is calculated. The output image displays the RGB overlay of the first three channels with the line position indicated and the corresponding intensity plots. The dots on the graphs mark the peak positions and the intensity values used for calculating the FWHM.

#### Field illumination

The image is first smoothened by a mean filter (radius 5 pixel) to reduce noise and to remove small artifacts due to dirt or scratches on the plastic slides. Then the horizontal and vertical intensity profiles through the centre of the image are measured [7,13]. The intensity profiles are further smoothened by reading out the average over 10 adjacent pixels (either in X or Y direction) rather than using a one pixel wide profile. The result image shows the smoothened and contrast enhanced field image with black lines marking where the intensity profiles were measured together with the intensity plotted versus pixel position. Minimum/maximum intensities and the percentage differences are calculated.

#### Bead Colocalisation

For the colocalisation analysis with the 1µm Tetraspeck beads the different channels are analysed separately [7,8,13]. First the images are median filtered (radius 7 pixel) to remove noise and to smoothen the image, then the brightest image in the stack is identified (typically the image with the maximum bead diameter). For the size or resolution measurements of beads one typically records the diameter obtained at the half maximal bead intensity (FWHM – full width at half maximum). We use this half-maximum intensity to set a channel specific threshold for the subsequent binarisation that is applied to the whole Z stack. The 3D centroid is calculated and the procedure repeated for the different channels. The image stack is also re-sliced in XZ and YZ direction at the centroid position. For visualization purposes the original images are overlaid as RGB images in XY, XZ and YZ with the Z views corrected (extended by the ratio of Z step size to XY pixel size) to obtain similar dimensions in XY and Z. The outlines of the thresholded images with the calculated centroids are presented in a similar fashion. The XY and Z distances between all centroids are calculated.

#### Point spread function

The PSF image stacks are processed similarly, but after the centroid has been calculated we obtain the intensity profiles at the centroid position in XY and Z direction and fit a Gaussian curve to obtain the lateral and axial FWHM as a measure for resolution [13,32,34]. For display purposes XY/XZ/YZ views are calculated and an image montage is created showing all the Z sections. To enhance the visibility of the weaker diffraction patterns the gamma setting is changed (0.1), contrast enhanced and a pseudo-colour look-up-table (LUT) applied.

#### Spectrophotometer accuracy

For the lambda scans the average image intensities are plotted versus the wavelength for all PMTs used [13,19]. The wavelengths corresponding to the peak intensities, the actual peak intensity and the FWHM are determined. The wavelengths of the three laser lines used are also indicated.

#### XY scanning galvos

The reflection image with the grid pattern is currently only contrast enhanced and saved. No further measurements of the square dimensions are implemented yet.

#### XY stage accuracy

The image series of the fluorescent beads are median filtered to reduce noise and binarised (threshold set to half of maximum pixel brightness, calculated for each image). The binary image is used as mask for the original image, creating a new image where only the above threshold intensities of the bead are displayed while all other pixels are set black (=0 intensity). Then XY centroid coordinates and average intensity of all pixels with values larger than 0 are calculated. The intensity measurements are useful to examine Z focus drift. To assess repeatability average and standard deviation for all the X and Y centroid positions are calculated and a plot is generated showing the scatter of the bead centroids around the average position. For the accuracy measurements we compare the stage movement as recorded in the metadata with the actual bead centroid movement within the same field of view. The actual distance traveled by the bead is calculated and subtracted from the stage movement. 

#### Z-galvo stability

To assess Z-galvo drift the Z position of the reflection peak is measured at each time point from the intensity profile of a vertical line running through the centre of the image from top to bottom. The peak position is plotted versus time [29]. Since vibrations during the course of a single Z scan can also affect the width of the main reflection peak the FWHM of the reflection peak is measured and plotted as a simple vibration indicator. 

## Results and Discussion

### How to use ConfocalCheck

**Figure 1:**
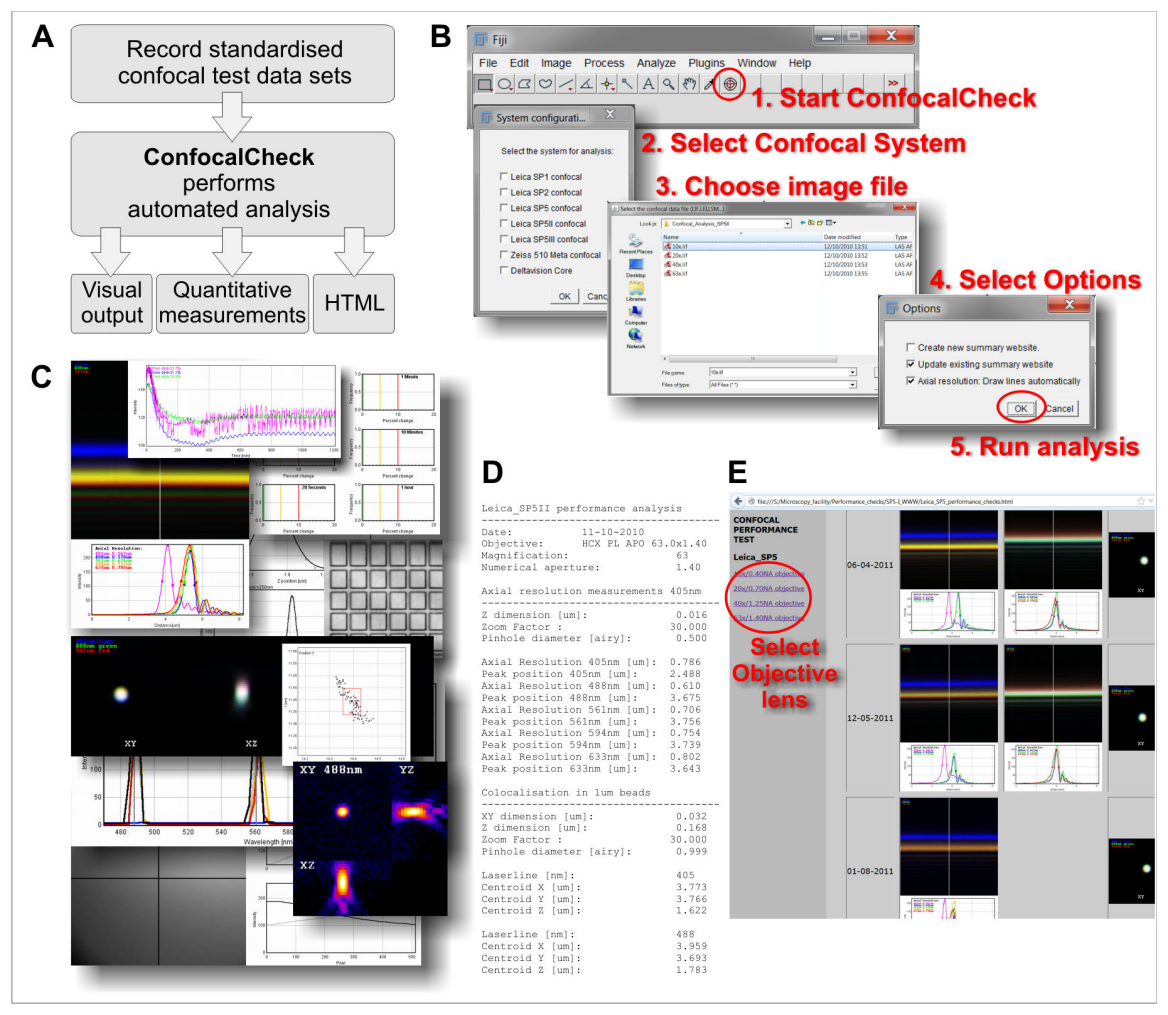
Using ConfocalCheck. **A**: Workflow of our approach. The analysis of the standardised test data with ConfocalCheck provides a range of numerical and visual outputs to monitor confocal performance. **B**: Running ConfocalCheck. Once installed in ImageJ/Fiji, click on the target pattern (step 1, marked by red circle) to start the software. Choose a confocal system (step 2), then the data files (step 3). The options window provides choices mainly related to the HTML output. Click OK to run the analysis. **C**: Collage of some of the output images created to provide quick visual feedback. **D**: The measurement file contains all the quantitative data used for a more detailed characterisation of the system. **E**: The HTML output is a very useful tool for long term record keeping and monitoring particularly in a facility environment with many confocal instruments. Different objective lenses are selected in the left frame of the browser window, the corresponding test data are displayed on the right.

ConfocalCheck was developed to assist with the qualitative and quantitative analysis of confocal microscope performance tests. Figure 1A shows the workflow of the proposed approach. First of all standardised test images are obtained. Image acquisition was simplified as much as possible to deliver reliable and reproducible test data sets (step-by-step instructions are available in Protocol S1). ConfocalCheck analyses the test images and provides a range of output files. ConfocalCheck is not platform specific - we have tested the software with Leica SP1/SP2/SP5 systems, a Zeiss LSM510 Meta and a LSM780 as well as a Nikon A1R confocal microscope. Data from other instruments can be imported as TIFF images.

ConfocalCheck is easy to use, the user interface has been kept very simple. Once the macro has been installed in ImageJ/Fiji a target pattern icon appears on the tool bar (Figure 1B). Click there to start ConfocalCheck. If there are multiple confocal systems, tick the appropriate box. Choose the test data file and select any options. On clicking OK the analysis will commence (for more details on how to use ConfocalCheck see Protocol S3). 

There are three different types of output data (Figure 1A). The images were designed to provide quick visual feedback on many important parameters (a subset of result images is shown in Figure 1C). The quantitative measurements are recorded in tab delimited text files for further analysis (Figure 1D) and the HTML output is an invaluable tool for record keeping (Figure 1E). We have used ConfocalCheck for two purposes: to monitor confocal performance and to compare individual components across different microscopes.

### Monitoring confocal performance

The main result of testing the confocal microscopes in our microscopy facility over the last three years was that these routine checks are absolutely essential to maintain system performance as many critical parameters changed over time. The supplementary text (Text S1) and figures (Figures S1-S11) provide detailed accounts of some of the issues we have encountered to illustrate this point. Most of these problems would not have been noticed by the average facility users with their biological samples. And the confocal microscope cannot be examined in isolation either, the impact of environmental factors like air flow and temperature changes also needs to be considered, for example by measuring room and/or microscope stage temperature in conjunction with some of the performance tests (Figure S2A/B, Figure S10B/C)[29,37].

Observing trends in the measurements - for example looking at the changes in laser power (Figure S1) or in the alignment of the spectral detectors (Figure S8C) - can help identifying potential issues which can be rectified before they cause problems for the users. The automated integration of the ConfocalCheck test results with the HTML web site format allows the operator to quickly browse through all the historic data and to spot those gradual changes that could easily be missed if one had to locate and compare the individual images.

Having done all the tests monthly for each system for about two years we re-assessed whether all of them were required in our setting as the recording of all the test data is time consuming. We have now an essential set of five tests: 

1. Maximum laser power. 2. Field illumination at 405nm. 3. Colocalisation of 1µm Tetraspeck beads. 4. Lambda scans (spectral sliders on Leica confocal systems). 5. Grid pattern (XY-scanning galvos). The 405nm field illumination and the colocalisation tests are mainly used to check the alignment of the 405nm laser optics, in particular the alignment of the objective specific 405nm correction lenses on our Leica SP5 systems (Fig. S4A/S5A). 

Other tests are not routinely performed any more except when problems are suspected. We stopped measuring the PSF as we didn’t observe any significant changes in the PSF shape or the derived resolution measurements (see Text S1, p. 8 )[27,32]. Although there are plenty of scratches and indentations on the front metal surface of the objective lenses indicating collisions with the metal sample holder and/or the motorised stages maybe the fact that our local users are not swapping or manually handling the objective lenses themselves prevents serious damage. The lens specific axial chromatic correction didn’t change either over time and is now only used for characterising new lenses.

Depending on the local instrument configuration, user needs and demands we would encourage users to choose a set of calibration checks and measure them routinely as good practice. As a minimum requirement the alignment and stability of the illuminating lasers, the detectors, optical performance of the objective lenses, the XY scanner and other important mechanical components of the microscope should be tested. To do nothing is not an option. Even with the most expensive service contract there is typically only one annual service visit by a confocal engineer which would not be sufficient as we have demonstrated. A misaligned system could have detrimental consequences for the acquired images as the observed fluorescence intensity patterns might not reflect the actual dye distribution in the specimen (Figures S4A-C, S5A-B, S9). The subsequent quantitative analysis or colocalisation measurements would also be affected in the worst case invalidating the conclusions drawn from these experiments.

### Using ConfocalCheck to compare microscope components

ConfocalCheck is also a useful tool to compare confocal instruments or individual components across multiple instruments as it provides the necessary quantitative measurements (Figure 1D). 

We examined in detail the chromatic correction of the objective lenses to illustrate the robustness and reproducibility of the metrics used (see Text S1; Figs. S5/S6). Our comparison of two different high performance plan-apochromat lenses from two different Leica microscopes clearly showed the similarity of equivalent lenses across the systems and as well as the differences between the lens types (Figs. S5C/S6D; for a similar comparison of 20× objective lenses on three instruments see Figs. S5D/S6A-B). 

Beyond this application, if equipment fails and another instrument needs to be used, if upgrades and changes are made to a piece of equipment, or if different objective lenses are tested before purchasing, the metrics provided by ConfocalCheck could be used for the necessary side by side comparisons. 

And if an equivalent objective lens in not available on the replacement instrument knowing the degree of bead displacement at a particular wavelength for example could help to correct this during post acquisition processing. To be able to reliably compare data that have been created over many months or years on the same instrument, or even to combine microscopic data from several laboratories is not trivial, it does require a significant and constant effort to ensure the performance of the confocal setup [10].

ConfocalCheck works well for the standardised data sets but is far from perfect. Anyone with some experience of the ImageJ macro language should be able to extend its capabilities or to adapt the code for specific instruments not currently covered. Significant improvements in speed and functionality could be achieved by rewriting this into a Java plugin for ImageJ and better integration with the LOCI Bioformats plugin [38] to use data from a wider range of microscope manufacturers. A more detailed analysis could be carried out to automatically assess the distortions of the square grid standards or to characterise the nature of the vibrations affecting the confocal setup [39].

Another tool for tracking confocal performance is MetroloJ [34], a freely available plugin developed for ImageJ [35]. It can analyse a limited range of important parameters (detector CV, field illumination, the psf [27], bead colocalisation, axial resolution) and extract very detailed quantitative metrics from the test images. Reports in the PDF file format and output images are created for documentation purposes. Some of the quantitative measurements are similar to the metrics provided by ConfocalCheck, there are however limitations. The colocalisation analysis is restricted to two or three channels, the axial resolution is only determined for one channel. As the user has to load the necessary data sets before starting MetroloJ and enter additional microscope information before the actual analysis MetroloJ would be more suitable for the detailed interactive analysis of smaller data sets while ConfocalCheck could be regarded as a batch processing tool for fully automated processing. 

With the increase in quantitative imaging and in medical applications of fluorescence based techniques regular performance checks with standardised samples and defined acquisition protocols will be key to ensure that appropriate experimental data are acquired [6,10]. Efforts are being made to develop suitable calibration and validation standards required to standardise microscopic imaging [6]. This is a major issue that needs to be addressed. Many confocal microscopes are not performing as well as they should as highlighted by a recent study comparing laser stability, field illumination and colocalisation on confocal systems from 23 laboratories spread across eight countries [37]. A large proportion of instruments failed the set acceptance criteria for the various tests. Some steps have been taken by manufacturers to simplify daily maintenance: Zeiss have introduced a special calibration objective lens used for the routine calibration of some instrument parameters like beam path alignment, pinhole adjustment and scanner calibration for their latest range of confocal microscopes. Leica offer a remote sensing system that monitors many instrument parameters for remote diagnostics and repair purposes. But although this could potentially provide valuable feedback directly to the microscope user or facility staff this is currently not implemented. Obtaining even very basic system information like regular laser power readings would be very helpful as our long term analysis has shown (Figure 1S). Nevertheless an independent evaluation of the instrument by the user is necessary and any issues uncovered need to be resolved in collaboration with the manufacturer. 

Our approach of combining standardised image acquisition with the easy-to-use ConfocalCheck analysis tool presented here should enable even inexperienced microscopists to evaluate and monitor the performance of their local confocal instrument. This is going to be essential given the increasing complexity and functionality of confocal microscopes that are now often combined with super-resolution techniques requiring even more stringent imaging conditions.

## Supporting Information

Figure S1 **Long term laser power variations.** **A**: Monthly maximum laser power measurements on a confocal microscope as recorded with a power meter. Arrows indicate an improvement in laser output following laser re-alignment by service engineers, “R” indicates the replacement of the Argon laser fibre, also in (B). **B**: Changing Argon laser power in a newly installed confocal microscope. The red 633nm HeNe and the 561nm DPSS lasers show a relatively stable output compared to the Argon laser.
(TIF)

Figure S2 **Short term laser fluctuations and the effect of room temperature.** **A**: Temporal changes in relative laser intensities correlate with changes in room temperature in approximately 30 minute cycles. Laser power was measured every 20 seconds with the transmitted light detector. The colours reflect the different laser lines. **B**: Intensity fluctuations on a confocal microscope in a different room with very small temperature changes. **C**: Overnight time course of a 405nm laser showing large erratic intensity variations compared to the stable 561nm line.
(TIF)

Figure S3 **Evaluating laser noise on a µs to hour time scale.** Detailed analysis of the transmitted light time-lapse recordings to assess laser noise. **A**: Readout of the laser intensity fluctuations along a single horizontal scan line plotted versus the pixel position, showing very little variation on. The colours reflect the different laser lines. **B**: Plotting the standard deviation of the pixel intensities of the whole image over time as a measure of laser intensity variation. The 594nm HeNe laser shows increased noise compared to the other lasers. **C/D**: Comparison of the intensity fluctuations of two Argon lasers over many different time scales relevant to typical scanning applications. The histograms show the pixel to pixel or image to image intensity differences (their relative frequencies) - depending on the time scale. Green bars indicate no variation. The laser in **D** shows larger intensity variations – increased noise - on short time scales within each frame while the image to image variation is similar to the laser in **C**.
(TIF)

Figure S4 **Field illumination.** **A**: A blue fluorescent plastic slide excited with the 405nm laser light through a 63×/1.40NA Plan-Apo lens. With this objective there should be little variation in intensity across the field, but there is significant misalignment. **B**: Pixel intensities along the black lines in **A**, displayed on two different intensity scales as smaller intensity variations were not always easy to spot on the full grey scale range. This simple readout together with the measured minimum and maximum values along the intensity profiles is useful to quantify the misalignment. **C**: Contrast enhanced image showing the effect of a dirty, probably oil contaminated tube lens on imaging a fluorescent test slide (HCX PL APO CS 100.0×/1.40NA). **D**: The faulty shutter of a 405nm laser opened late while already scanning the test slide causing the dark strip across the top of the image (marked by red arrows; 20×/0.70NA HC PL APO CS).
(TIF)

Figure S5 **Colocalisation analysis using fluorescent beads.** **A**: Confocal sections of a 1µm fluorescent Tetraspeck bead recorded with a Leica 40×/1.25NA oil HCX PL APO CS objective lens and the indicated laser lines. The bead image (original) shows the RGB overlay of the channels recorded with 405/488/561nm excitation, the image below the outlines of the thresholded bead images for all 4 wavelengths. The black outline marks the 633nm excitation. These plots are created automatically by the ConfocalCheck macro as well as the XYZ centroid positions and the pairwise centroid distances for the different excitation wavelengths shown in C/D. The complete axial displacement of the blue image is due to the use of the wrong 405nm correction lens. The arrows indicate the lateral misalignment of the 405nm channel. **B**: Overlay images of 1µm Tetraspeck beads recorded with a 10×/0.30NA dry HC PL FLUOTAR and a 40×/1.25NA oil HCX PL APO CS lens before and after the confocal scanhead was properly attached to the microscope stand. **C**: Comparison of the pairwise XY and Z centroid distances between the 40×/1.25NA oil HCX PL APO and 63×/1.40NA oil HCX PL APO lambda blue from two different confocal microscopes. We analysed the centroid distances obtained from images acquired with the 488/561nm excitation and with the 488/633nm pair. While the XY distances are very small (50-100nm) and very similar for all the lenses, there are clear differences between the two lens types in Z direction. **D**: Comparison of the pairwise XYZ centroid distances between the 20× objectives available on three different Leica SP5 systems (20×/0.50NA HCX PL FLUOTAR vs 20×/0.70NA HC PL APO CS). There are slight differences in the laser configuration as indicated (561nm vs 543nm). The fluorite FLUOTAR is less well corrected for the red/far-red part of the spectrum compared to the plan-apochromatic lens, causing significant displacement of the centroid/bead image in the Z-direction.
(TIF)

Figure S6 **Comparing the axial chromatic correction of objective lenses using reflected light.** **A**: Overlay of three XZ scans recorded with 488/543/ 633nm laser light in reflection mode. Objective: Leica HCX PL APO CS 20×/0.70NA multi-immersion. The graph shows the intensity profile along the white line in the image indicating good overlap and chromatic correction. **B**: Overlay of the three XZ scans recorded with a less well corrected Leica HC PL FLUOTAR 20×/0.50 NA lens available on the same microscope. The arrows indicate the displacement of the 633nm reflection band. **C**: Comparing the axial chromatic correction of three different lenses by plotting the positions of the peak reflections obtained for the various laser lines relative to the position of the 458nm peak. The laser lines are: 458/488/514/561/594/633nm. Objectives (all Leica): 10×/0.30NA dry HC PL FLUOTAR, 10×/0.40NA HCX PL APO CS, 20×/0.70NA HC PL APO CS. **D**: The axial chromatic correction of different oil immersion lenses. Objectives (all Leica): 40×/1.25NA HCX PL APO CS, 63×/1.40NA oil HCX PL APO lambda blue, 100×/1.44NA oil HCX PL APO CS. **E**: Axial resolution measured as the FWHM of the 488nm reflection band. Same objectives as in **D**. Resolution depending on the objective lens and pinhole diameter.
(TIF)

Figure S7 **Analysing the point spread function.** **A**: Different views/sections of the point spread function recorded with green 175nm PS speck beads and a 63×/1.40NA oil lens. XY: brightest section from the image stack; XZ/YZ corresponding views in the Z direction at the centroid position. These images were stretched in Z direction to match the lateral resolution. **B**: Measuring the lateral and axial resolution (FWHM) from the fitted gaussian curves. **C**: Corresponding montage of the individual Z stack images. A pseudo-colour LUT was applied to enhance the visibility of low intensity diffraction patterns.
(TIF)

Figure S8 **Testing the spectrophotometer accuracy on Leica SP systems.** **A**: A wavelength/lambda scan was carried out with a 5nm wide detection window over a 200nm range for each detector measuring the reflection of three laser lines from a mirror slide. The average image intensities were plotted versus the wavelength. The three peaks occur due to the reflection of the laser light at these wavelengths, except for PMT5 showing very little response (arrow) due to a faulty spectral slider unit. **B**: Very broad detection peaks for PMT1 indicating problems with the movement of the mirrors on the spectral slider unit. **C**: Gradual loss of the 633nm laser reflection over time on another system, restored in October following re-calibration for that wavelength.
(TIF)

Figure S9 **Image distortions.** Image distortions due to issues with the X and Y scanning galvos revealed by imaging a reflective square grid pattern. **A**: Stretched squares at the top of the image implicating the Y-scanning galvo. **B**: Distortions of the grid in X-direction as indicated by the two red squares.
(TIF)

Figure S10 **Microscope stage stability and the effects of temperature.** **A**: Stage Z drift. XZ time lapse recordings were carried out using the Z-galvo of a Leica SP5 system imaging the reflection of laser light from a mirror slide. The Z position of the reflection intensity peak is plotted versus time indicating a Z drift of 2.4µm in 60minutes. **B**: The inset shows successive images of the reflection bands with their width changing over time (2s intervals). The graph plots the FWHM for all time points (same data set as in **A**) showing little variation. Large erratic fluctuations of the FWHM can result from vibrations affecting the imaging system.
**C**: The effect of temperature on stage stability. XZ scans were performed while measuring the stage temperature at the same time. Air temperature in the environmental chamber surrounding the microscope was 37°C. The grey curve shows the temperature obtained with the probe, measured in 0.0625°C steps. The small temperature changes are just about resolved. A clearer signal was obtained by calculating a moving average over 60 seconds revealing the ~30min period caused by the air conditioning unit (black curve). The red curve indicates the corresponding focus changes as derived from the Z-position of the reflection peak signal.
(TIF)

Figure S11 **Testing the performance of motorised stages.** **A**: Centroid position of a fluorescent 1µm bead showing extensive drift of the motorised stage on this microscope during the course of a 2 hour time lapse experiment. The inset shows bead movement after the faulty stage was replaced (grey: individual centroid positions, red: average ± standard deviation). **B**: Repeatability of stage movement. The positions of three different beads on the microscope slide were repeatedly visited and imaged (100 times) and the average bead centroids determined. This was done twice for each of the two stages tested (see colours). The motorised stages moved back to the same positions within 0.5 to 1µm.
(TIF)

Protocol S1 **Image Acquisition.** Step-by-step instructions describing the acquisition of the confocal test images that can be analysed with the ConfocalCheck macro.
(DOC)

Protocol S2 **Editing the System Configuration.** Instructions on how to edit the system configuration file required by the ConfocalCheck macro. The configuration file "ConfocalCheck_Configuration.txt" contains information about the objective lenses and the laser lines used for the various assays.
(DOCX)

Protocol S3 **Using ConfocalCheck.** Step-by-step instructions describing the use of the ConfocalCheck macro, also containing details of all the output files created by the software.
(DOCX)

Macro S1 **ConfocalCheck.** Source code of the ConfocalCheck macro. It is used in conjunction with the free ImageJ or Fiji software (see Protocol S3 Using ConfocalCheck).
(TXT)

File S1 **Sample ConfocalCheck Configuration file.** This ConfocalCheck configuration file can be used to analyse the supplementary test data sets (Files S2, S3, and S4). It contains several example configurations that would have to be edited to match the local microscope configurations (see Protocol S2 Editing the System Configuration). The file has to be saved in the ImageJ\macros or Fiji.app\macros folder as “ConfocalCheck_Configuration.txt”. The ConfocalCheck macro will display an error message if the configuration file is missing.
(TXT)

File S2 **Test Dataset LeicaSP5II_10x.** Compressed ZIP archive containing test images recorded on a Leica SP5II confocal microscope using a 10×objective lens. Unpack and save the “Dataset_S6_LeicaSP5II_10x.lif” file. Analyse the images as described in “Protocol S3 Using ConfocalCheck”.
(ZIP)

File S3 **Test Dataset LeicaSP5II_20x.** Compressed ZIP archive containing test images recorded on a Leica SP5II confocal microscope using a 20×objective lens. Unpack and save the “Dataset_S7_LeicaSP5II_20x.lif” file. Analyse the images as described in “Protocol S3 Using ConfocalCheck”.
(ZIP)

File S4 **Test Dataset LeicaSP5II_63x.** Compressed ZIP archive containing test images recorded on a Leica SP5II confocal microscope using a 63×objective lens. Unpack and save the “Dataset_S8_LeicaSP5II_63x.lif” file. Analyse the images as described in “Protocol S3 Using ConfocalCheck”.
(ZIP)

Text S1 **The supplementary text describes in detail the type of analysis that can be carried out when using ConfocalCheck with the standardised test data sets.**
(DOC)
