# Supplementary figures and images for: ConfocalCheck - A Software Tool for the Automated Monitoring of Confocal Microscope Performance
Source: PLoS One. 2013 Nov 5;8(11):e79879. doi: 10.1371/journal.pone.0079879 (PMC3818239; doi:10.1371/journal.pone.0079879)

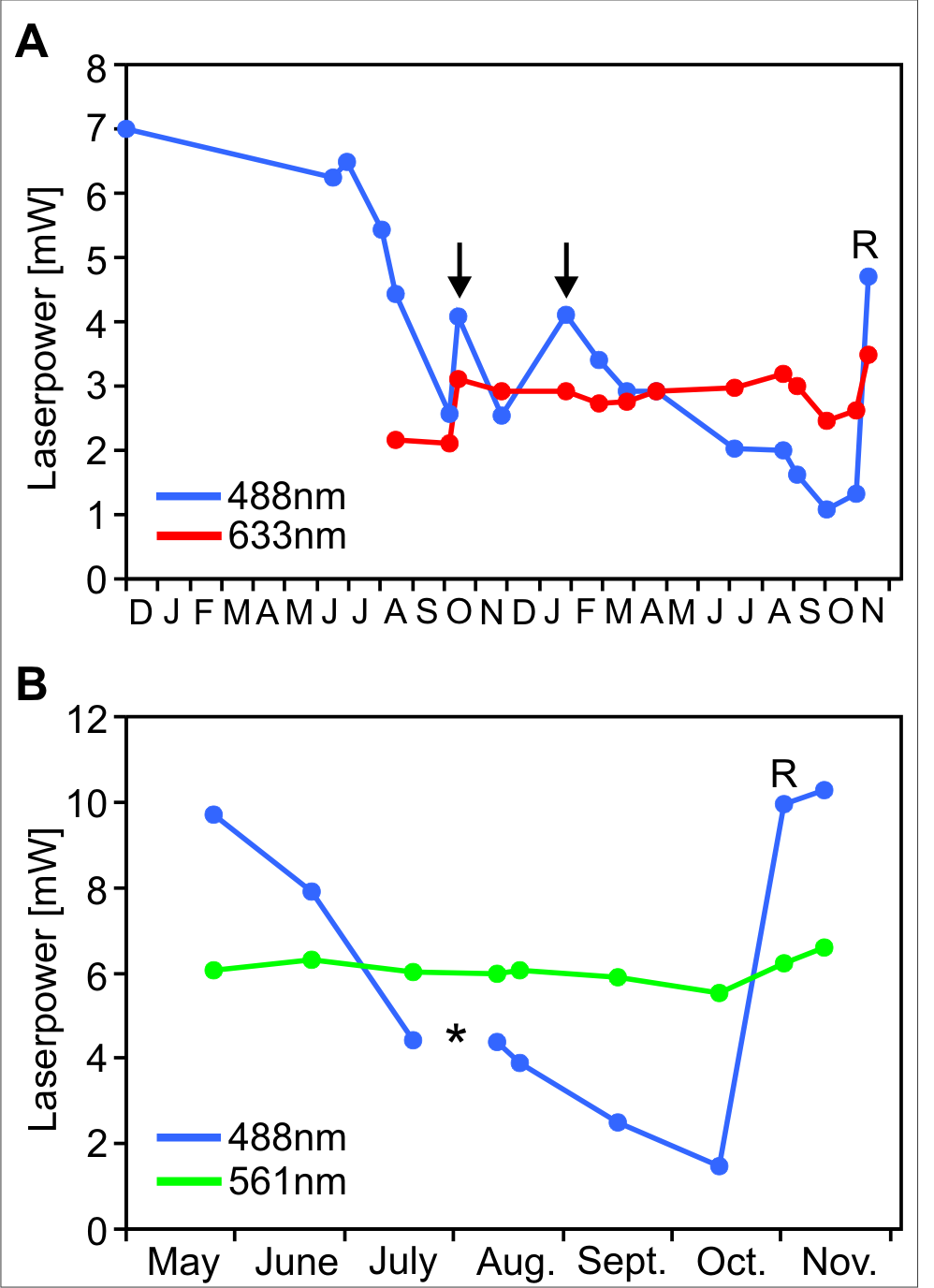

Supplement: Figure S1 — Long term laser power variations. A: Monthly maximum laser power measurements on a confocal microscope as recorded with a power meter. Arrows indicate an improvement in laser output following laser re-alignment by service engineers, “R” indicates the replacement of the Argon laser fibre, also in (B). B: Changing Argon laser power in a newly installed confocal microscope. The red 633nm HeNe and the 561nm DPSS lasers show a relatively stable output compared to the Argon laser. (TIF) [file pone.0079879.s001.tif]

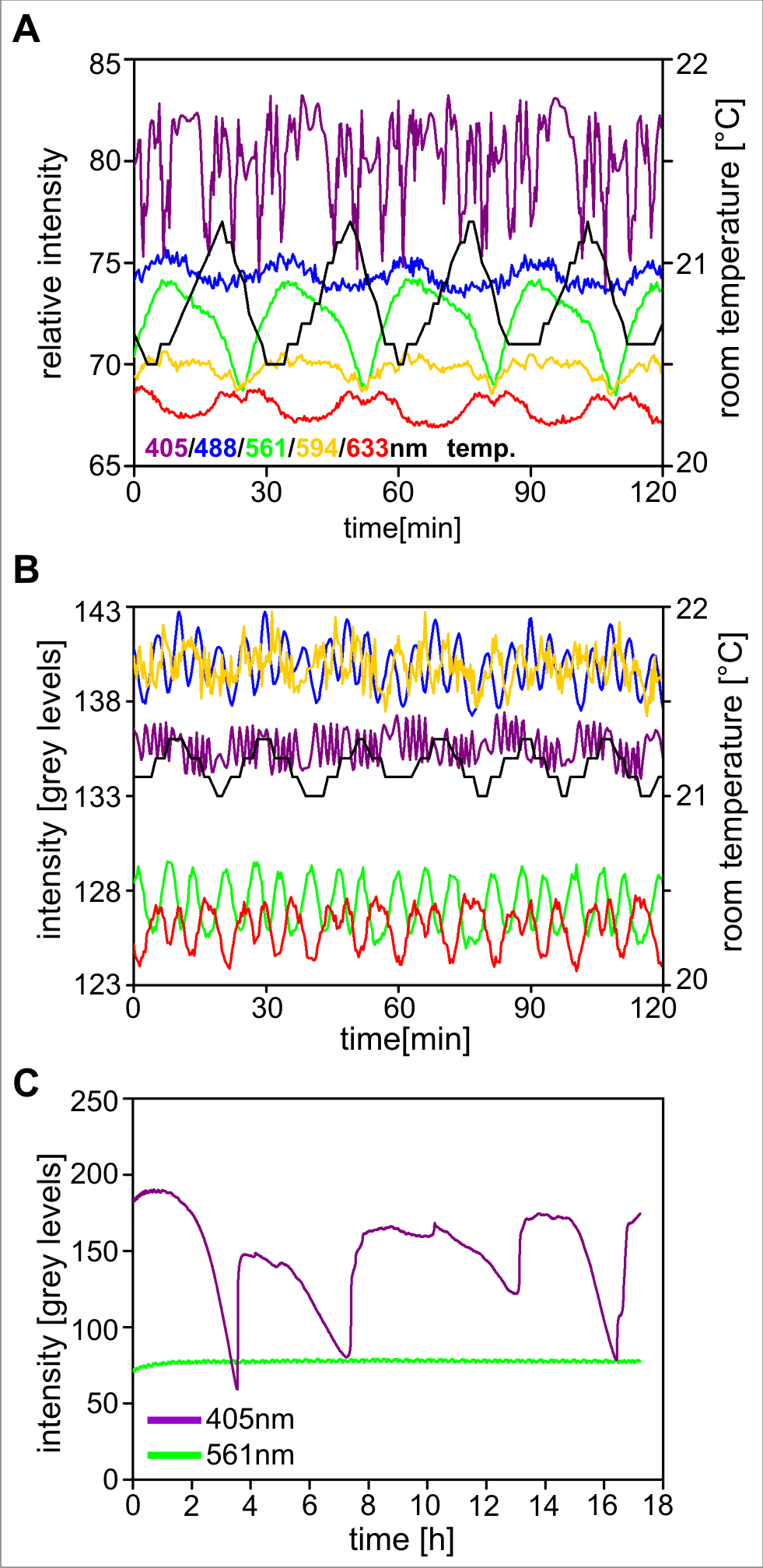

Supplement: Figure S2 — Short term laser fluctuations and the effect of room temperature. A: Temporal changes in relative laser intensities correlate with changes in room temperature in approximately 30 minute cycles. Laser power was measured every 20 seconds with the transmitted light detector. The colours reflect the different laser lines. B: Intensity fluctuations on a confocal microscope in a different room with very small temperature changes. C: Overnight time course of a 405nm laser showing large erratic intensity variations compared to the stable 561nm line. (TIF) [file pone.0079879.s002.tif]

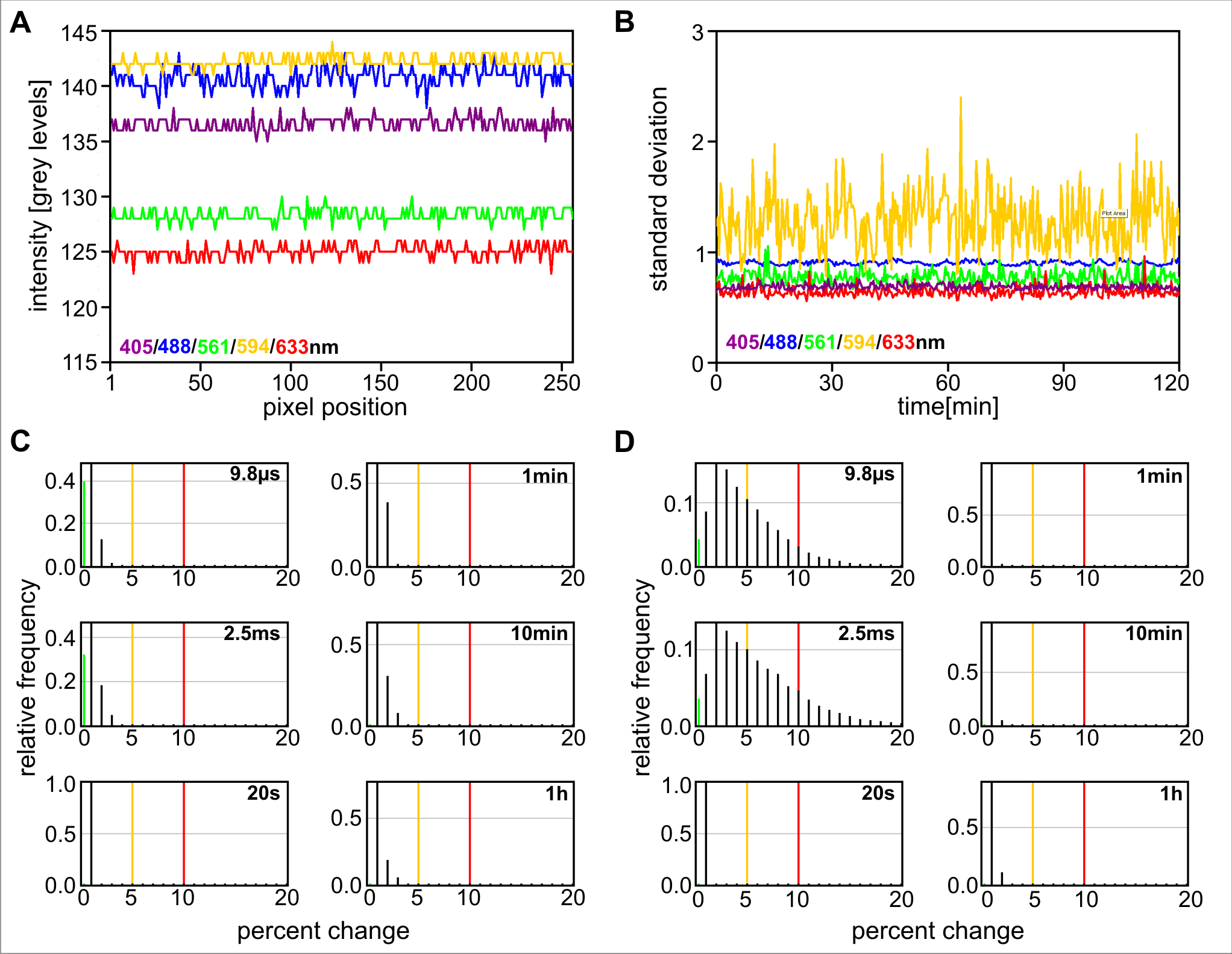

Supplement: Figure S3 — Evaluating laser noise on a µs to hour time scale. Detailed analysis of the transmitted light time-lapse recordings to assess laser noise. A: Readout of the laser intensity fluctuations along a single horizontal scan line plotted versus the pixel position, showing very little variation on. The colours reflect the different laser lines. B: Plotting the standard deviation of the pixel intensities of the whole image over time as a measure of laser intensity variation. The 594nm HeNe laser shows increased noise compared to the other lasers. C/D: Comparison of the intensity fluctuations of two Argon lasers over many different time scales relevant to typical scanning applications. The histograms show the pixel to pixel or image to image intensity differences (their relative frequencies) - depending on the time scale. Green bars indicate no variation. The laser in D shows larger intensity variations – increased noise - on short time scales within each frame while the image to image variation is similar to the laser in C. (TIF) [file pone.0079879.s003.tif]

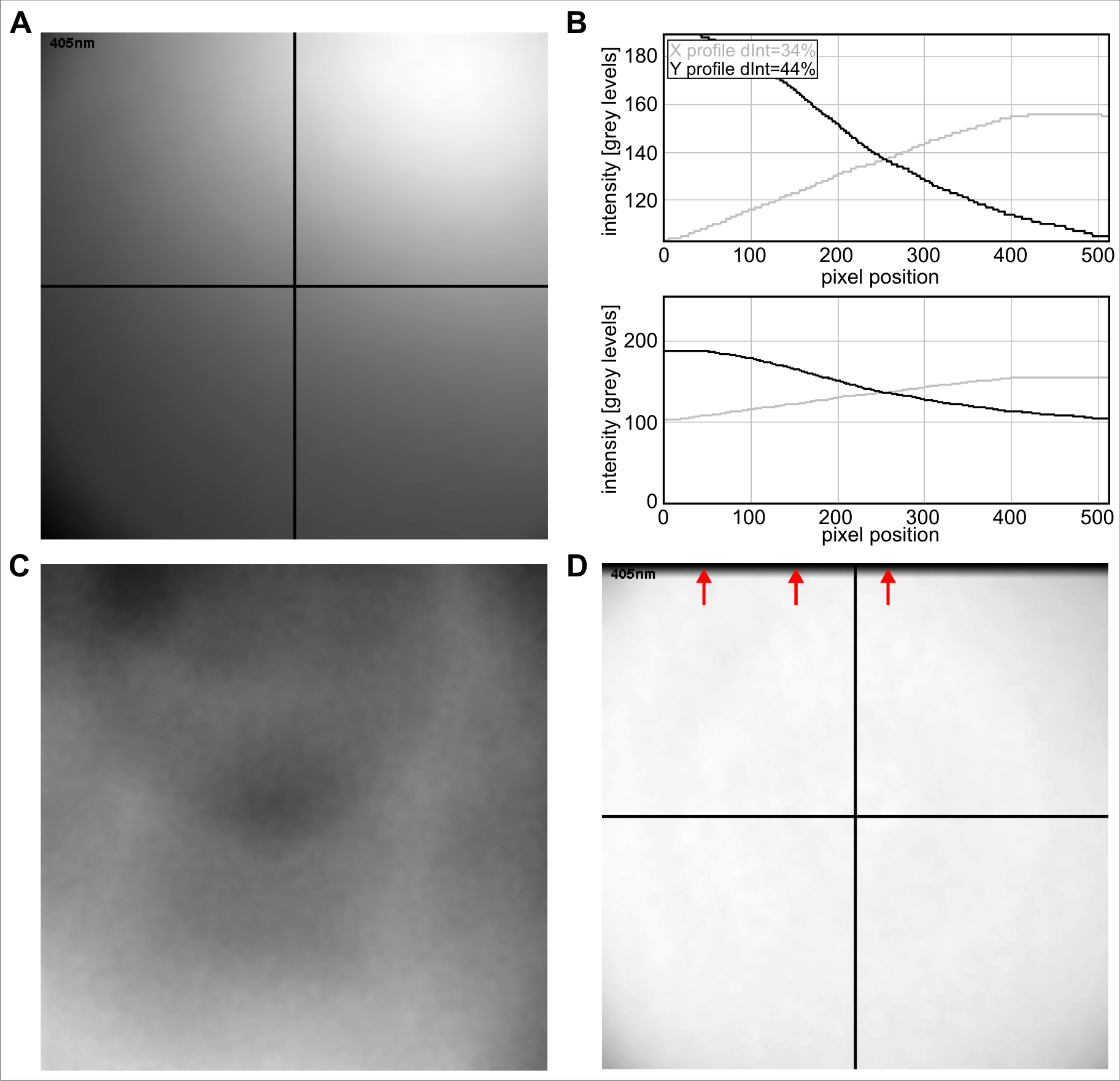

Supplement: Figure S4 — Field illumination. A: A blue fluorescent plastic slide excited with the 405nm laser light through a 63×/1.40NA Plan-Apo lens. With this objective there should be little variation in intensity across the field, but there is significant misalignment. B: Pixel intensities along the black lines in A, displayed on two different intensity scales as smaller intensity variations were not always easy to spot on the full grey scale range. This simple readout together with the measured minimum and maximum values along the intensity profiles is useful to quantify the misalignment. C: Contrast enhanced image showing the effect of a dirty, probably oil contaminated tube lens on imaging a fluorescent test slide (HCX PL APO CS 100.0×/1.40NA). D: The faulty shutter of a 405nm laser opened late while already scanning the test slide causing the dark strip across the top of the image (marked by red arrows; 20×/0.70NA HC PL APO CS). (TIF) [file pone.0079879.s004.tif]

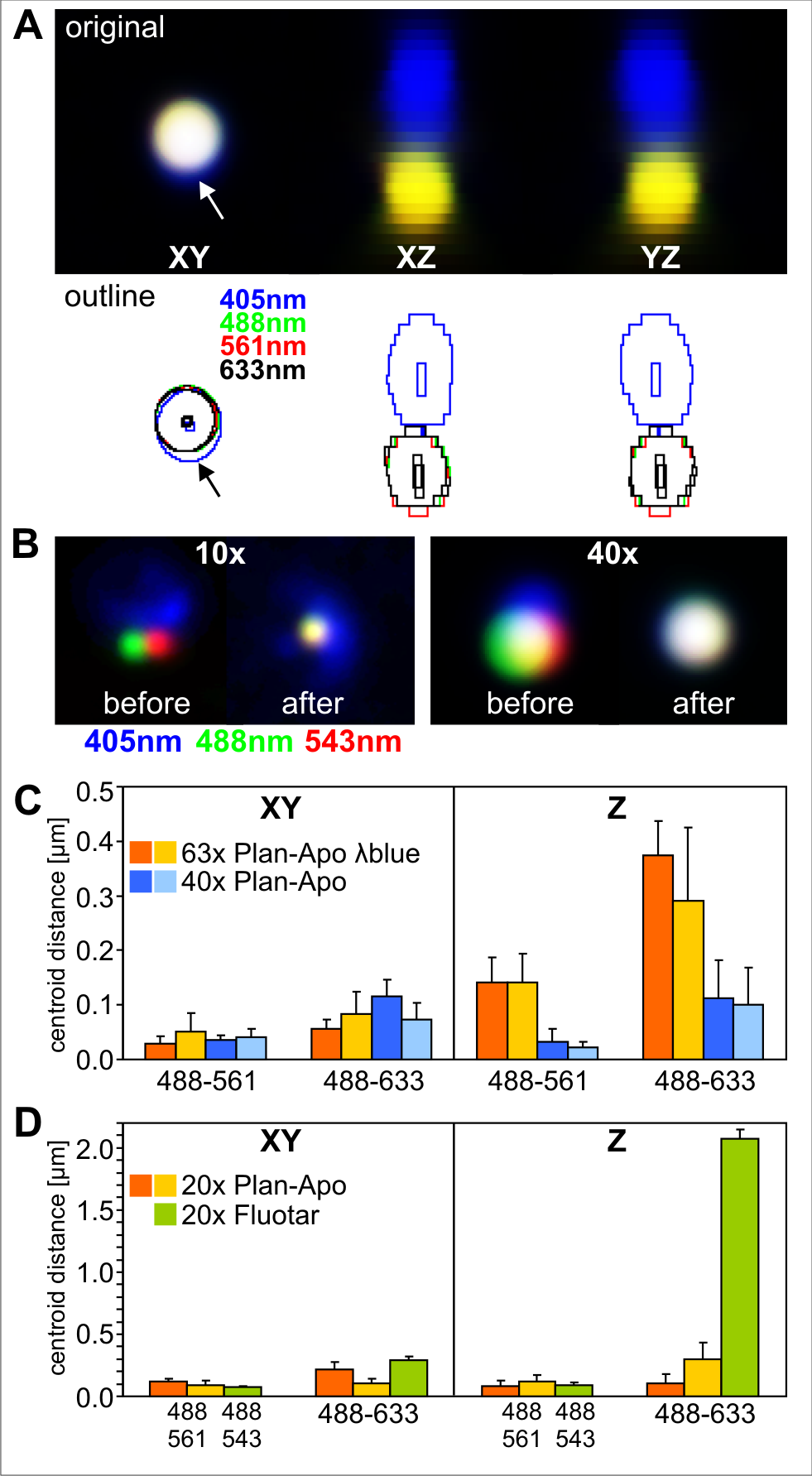

Supplement: Figure S5 — Colocalisation analysis using fluorescent beads. A: Confocal sections of a 1µm fluorescent Tetraspeck bead recorded with a Leica 40×/1.25NA oil HCX PL APO CS objective lens and the indicated laser lines. The bead image (original) shows the RGB overlay of the channels recorded with 405/488/561nm excitation, the image below the outlines of the thresholded bead images for all 4 wavelengths. The black outline marks the 633nm excitation. These plots are created automatically by the ConfocalCheck macro as well as the XYZ centroid positions and the pairwise centroid distances for the different excitation wavelengths shown in C/D. The complete axial displacement of the blue image is due to the use of the wrong 405nm correction lens. The arrows indicate the lateral misalignment of the 405nm channel. B: Overlay images of 1µm Tetraspeck beads recorded with a 10×/0.30NA dry HC PL FLUOTAR and a 40×/1.25NA oil HCX PL APO CS lens before and after the confocal scanhead was properly attached to the microscope stand. C: Comparison of the pairwise XY and Z centroid distances between the 40×/1.25NA oil HCX PL APO and 63×/1.40NA oil HCX PL APO lambda blue from two different confocal microscopes. We analysed the centroid distances obtained from images acquired with the 488/561nm excitation and with the 488/633nm pair. While the XY distances are very small (50-100nm) and very similar for all the lenses, there are clear differences between the two lens types in Z direction. D: Comparison of the pairwise XYZ centroid distances between the 20× objectives available on three different Leica SP5 systems (20×/0.50NA HCX PL FLUOTAR vs 20×/0.70NA HC PL APO CS). There are slight differences in the laser configuration as indicated (561nm vs 543nm). The fluorite FLUOTAR is less well corrected for the red/far-red part of the spectrum compared to the plan-apochromatic lens, causing significant displacement of the centroid/bead image in the Z-direction. (TIF) [file pone.0079879.s005.tif]

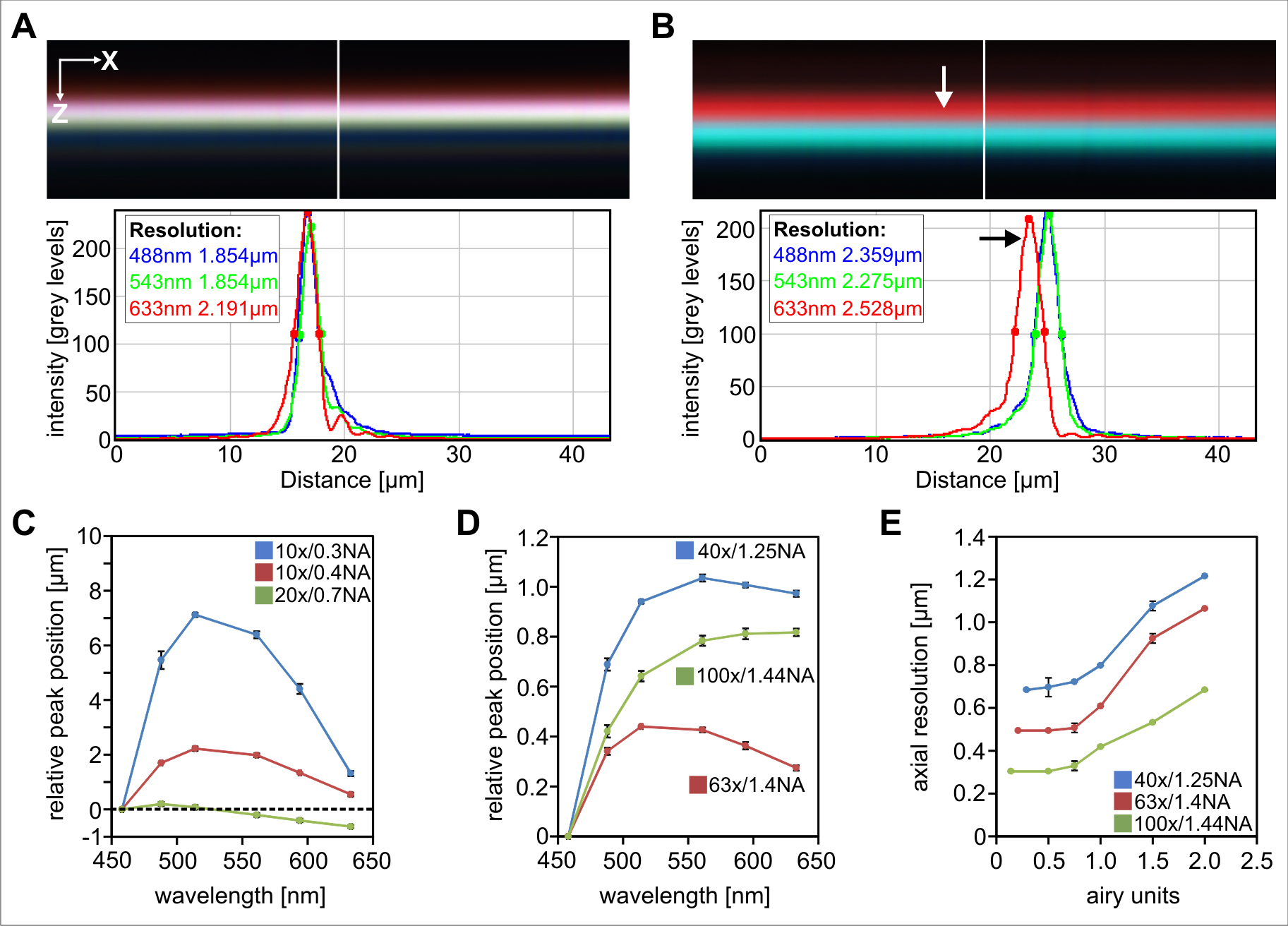

Supplement: Figure S6 — Comparing the axial chromatic correction of objective lenses using reflected light. A: Overlay of three XZ scans recorded with 488/543/ 633nm laser light in reflection mode. Objective: Leica HCX PL APO CS 20×/0.70NA multi-immersion. The graph shows the intensity profile along the white line in the image indicating good overlap and chromatic correction. B: Overlay of the three XZ scans recorded with a less well corrected Leica HC PL FLUOTAR 20×/0.50 NA lens available on the same microscope. The arrows indicate the displacement of the 633nm reflection band. C: Comparing the axial chromatic correction of three different lenses by plotting the positions of the peak reflections obtained for the various laser lines relative to the position of the 458nm peak. The laser lines are: 458/488/514/561/594/633nm. Objectives (all Leica): 10×/0.30NA dry HC PL FLUOTAR, 10×/0.40NA HCX PL APO CS, 20×/0.70NA HC PL APO CS. D: The axial chromatic correction of different oil immersion lenses. Objectives (all Leica): 40×/1.25NA HCX PL APO CS, 63×/1.40NA oil HCX PL APO lambda blue, 100×/1.44NA oil HCX PL APO CS. E: Axial resolution measured as the FWHM of the 488nm reflection band. Same objectives as in D. Resolution depending on the objective lens and pinhole diameter. (TIF) [file pone.0079879.s006.tif]

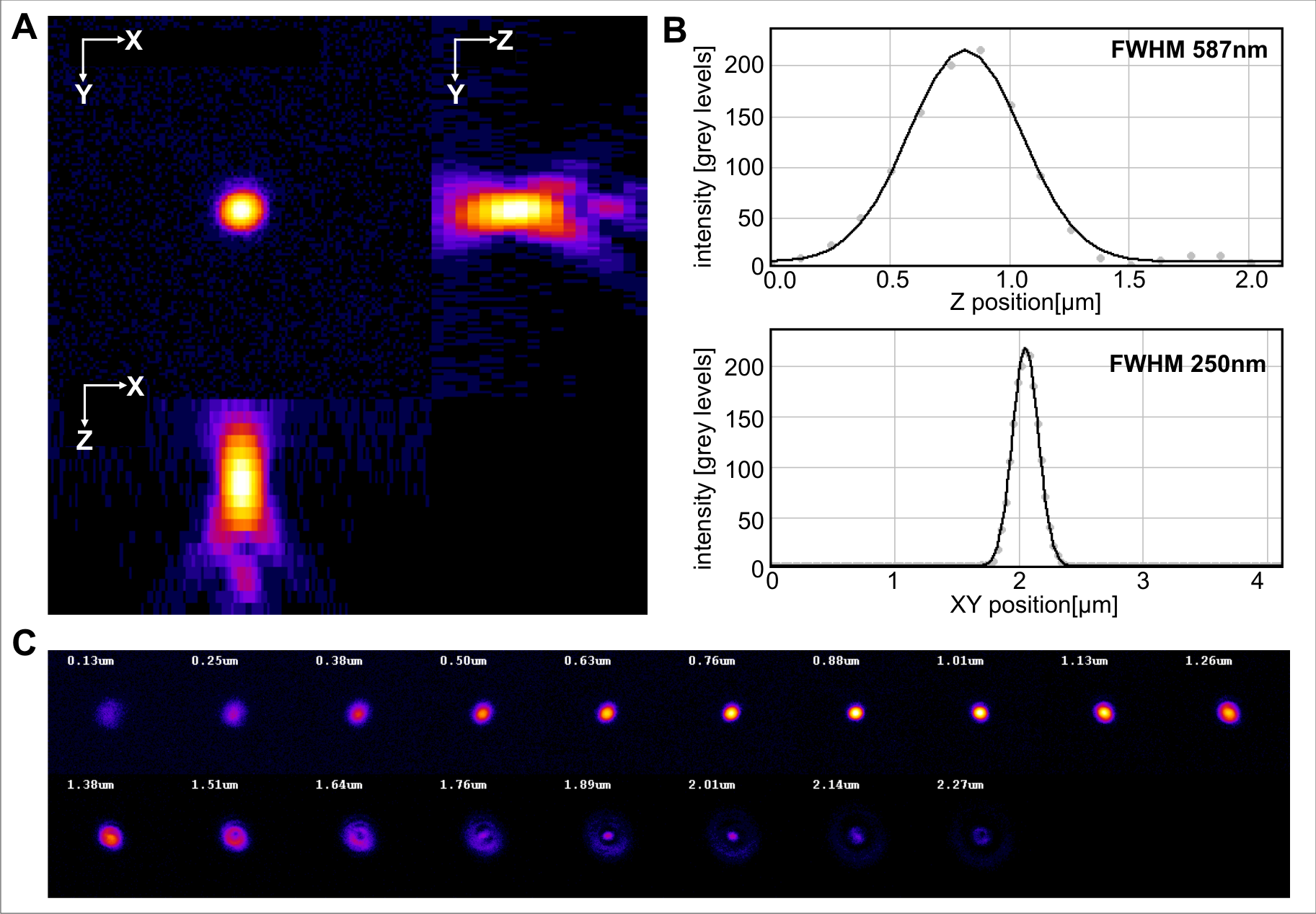

Supplement: Figure S7 — Analysing the point spread function. A: Different views/sections of the point spread function recorded with green 175nm PS speck beads and a 63×/1.40NA oil lens. XY: brightest section from the image stack; XZ/YZ corresponding views in the Z direction at the centroid position. These images were stretched in Z direction to match the lateral resolution. B: Measuring the lateral and axial resolution (FWHM) from the fitted gaussian curves. C: Corresponding montage of the individual Z stack images. A pseudo-colour LUT was applied to enhance the visibility of low intensity diffraction patterns. (TIF) [file pone.0079879.s007.tif]

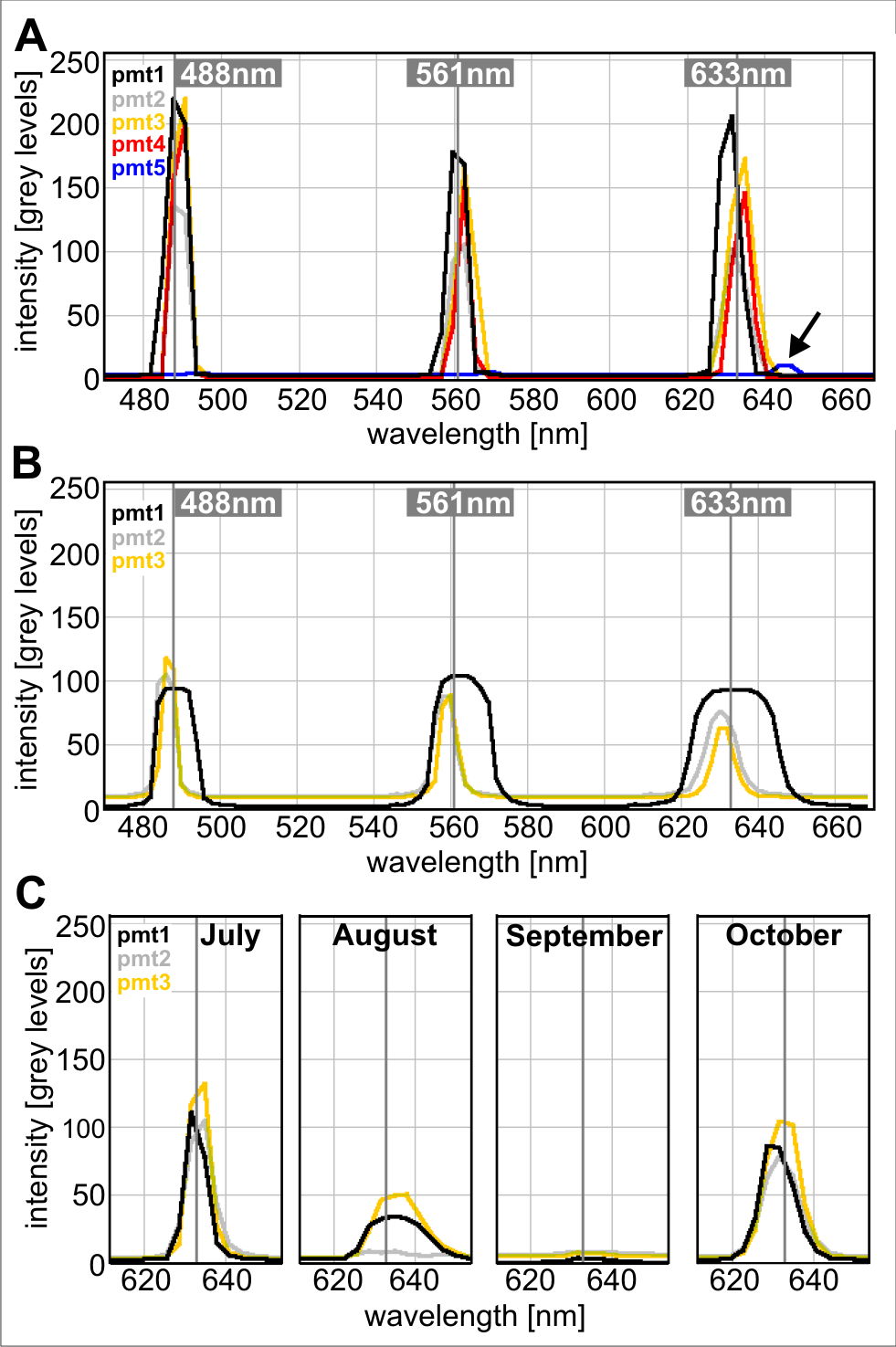

Supplement: Figure S8 — Testing the spectrophotometer accuracy on Leica SP systems. A: A wavelength/lambda scan was carried out with a 5nm wide detection window over a 200nm range for each detector measuring the reflection of three laser lines from a mirror slide. The average image intensities were plotted versus the wavelength. The three peaks occur due to the reflection of the laser light at these wavelengths, except for PMT5 showing very little response (arrow) due to a faulty spectral slider unit. B: Very broad detection peaks for PMT1 indicating problems with the movement of the mirrors on the spectral slider unit. C: Gradual loss of the 633nm laser reflection over time on another system, restored in October following re-calibration for that wavelength. (TIF) [file pone.0079879.s008.tif]

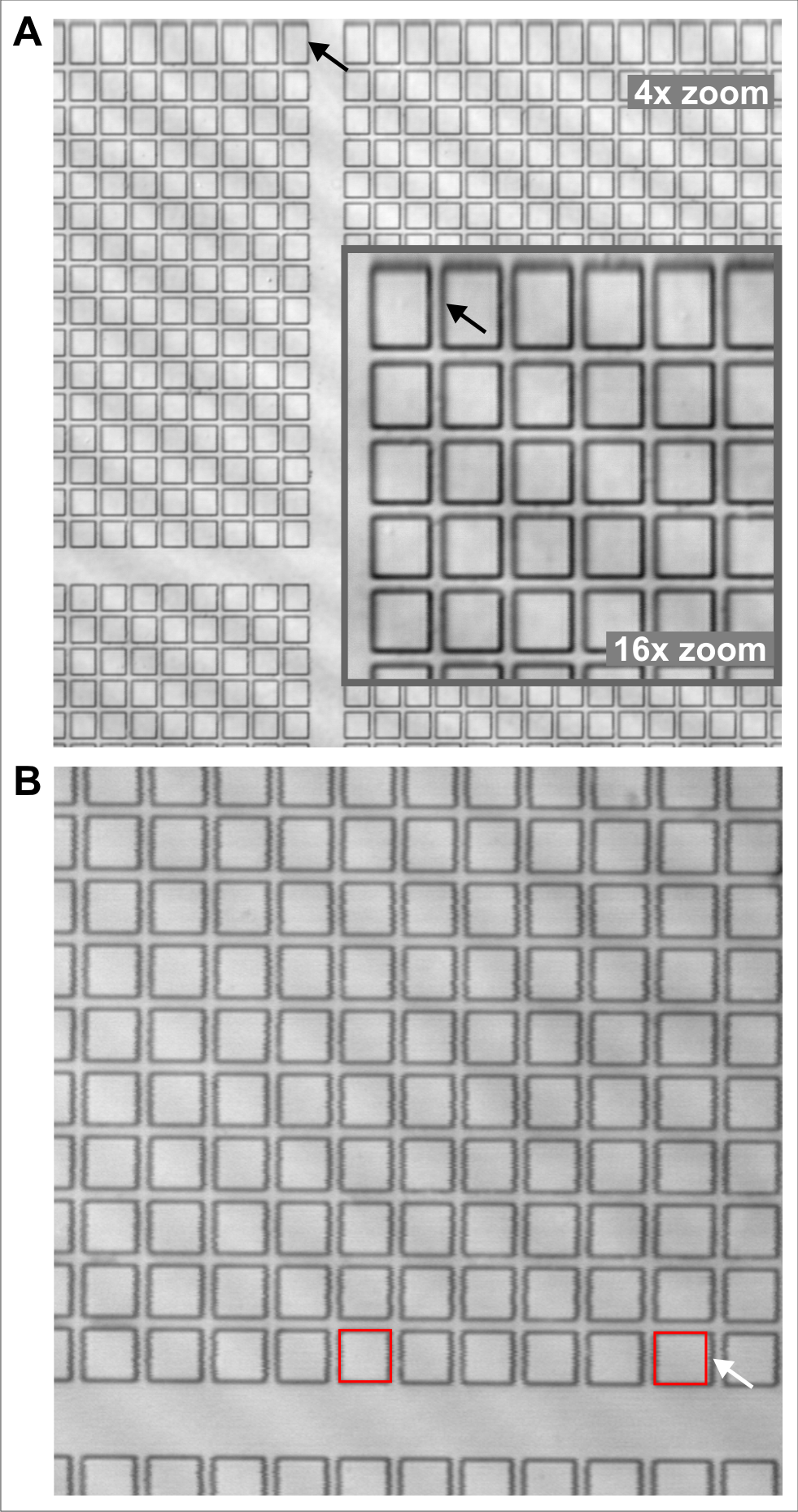

Supplement: Figure S9 — Image distortions. Image distortions due to issues with the X and Y scanning galvos revealed by imaging a reflective square grid pattern. A: Stretched squares at the top of the image implicating the Y-scanning galvo. B: Distortions of the grid in X-direction as indicated by the two red squares. (TIF) [file pone.0079879.s009.tif]

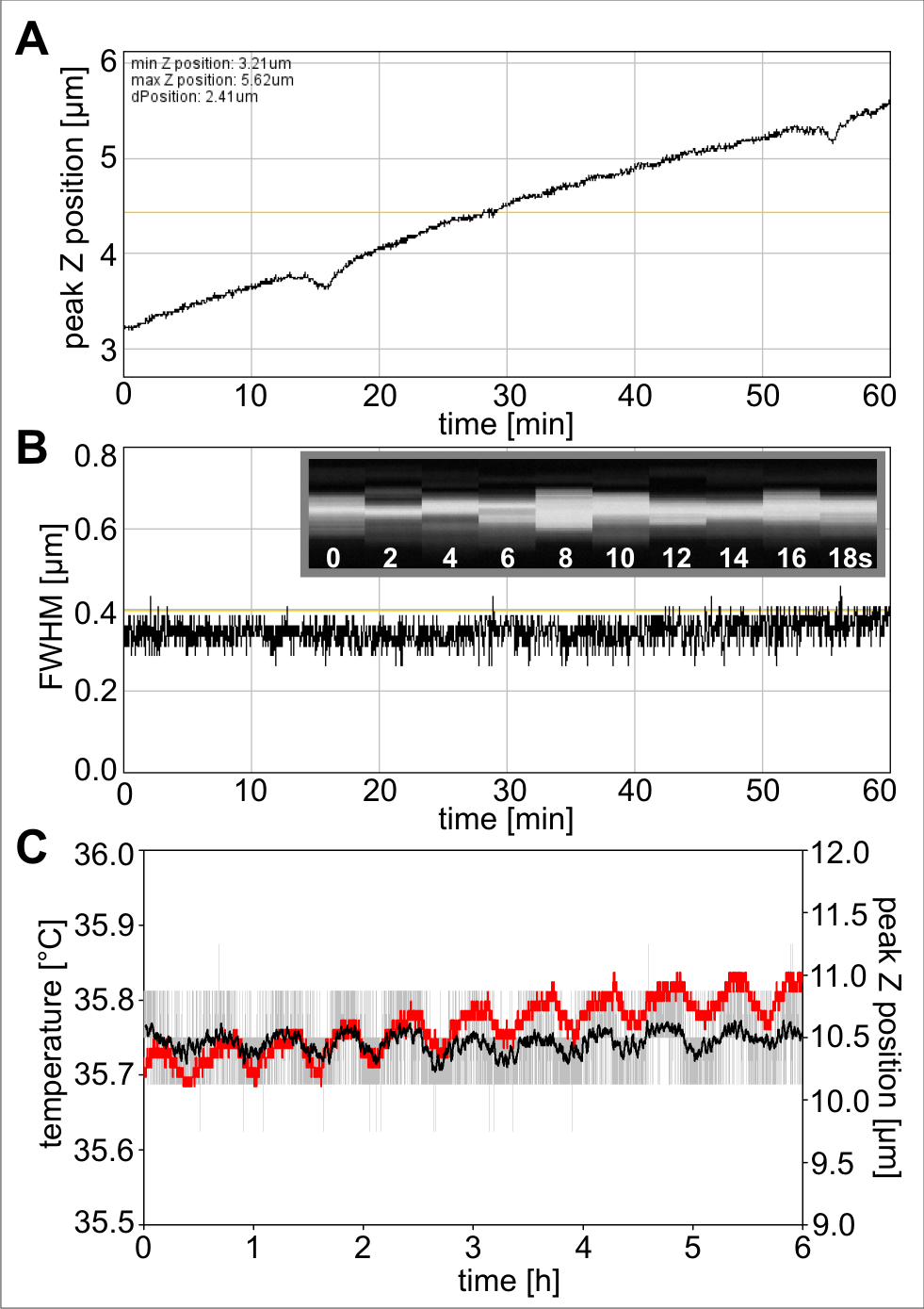

Supplement: Figure S10 — Microscope stage stability and the effects of temperature. A: Stage Z drift. XZ time lapse recordings were carried out using the Z-galvo of a Leica SP5 system imaging the reflection of laser light from a mirror slide. The Z position of the reflection intensity peak is plotted versus time indicating a Z drift of 2.4µm in 60minutes. B: The inset shows successive images of the reflection bands with their width changing over time (2s intervals). The graph plots the FWHM for all time points (same data set as in A) showing little variation. Large erratic fluctuations of the FWHM can result from vibrations affecting the imaging system. C: The effect of temperature on stage stability. XZ scans were performed while measuring the stage temperature at the same time. Air temperature in the environmental chamber surrounding the microscope was 37°C. The grey curve shows the temperature obtained with the probe, measured in 0.0625°C steps. The small temperature changes are just about resolved. A clearer signal was obtained by calculating a moving average over 60 seconds revealing the ~30min period caused by the air conditioning unit (black curve). The red curve indicates the corresponding focus changes as derived from the Z-position of the reflection peak signal. (TIF) [file pone.0079879.s010.tif]

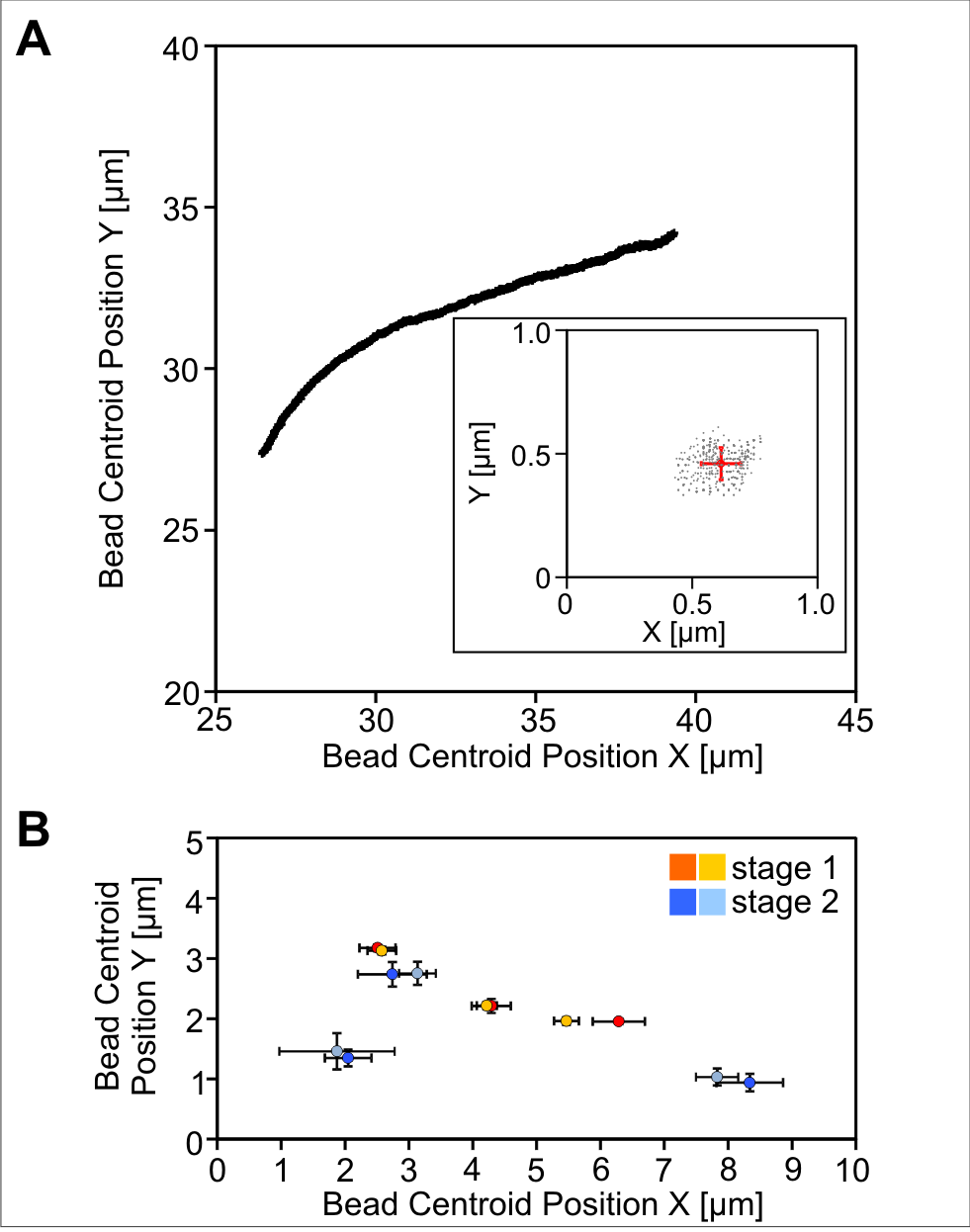

Supplement: Figure S11 — Testing the performance of motorised stages. A: Centroid position of a fluorescent 1µm bead showing extensive drift of the motorised stage on this microscope during the course of a 2 hour time lapse experiment. The inset shows bead movement after the faulty stage was replaced (grey: individual centroid positions, red: average ± standard deviation). B: Repeatability of stage movement. The positions of three different beads on the microscope slide were repeatedly visited and imaged (100 times) and the average bead centroids determined. This was done twice for each of the two stages tested (see colours). The motorised stages moved back to the same positions within 0.5 to 1µm. (TIF) [file pone.0079879.s011.tif]
