# Supplementary material for: ConfocalCheck - A Software Tool for the Automated Monitoring of Confocal Microscope Performance
Source: PLoS One. 2013 Nov 5;8(11):e79879. doi: 10.1371/journal.pone.0079879 (PMC3818239; doi:10.1371/journal.pone.0079879)
Supplement: Protocol S1 — Image Acquisition. Step-by-step instructions describing the acquisition of the confocal test images that can be analysed with the ConfocalCheck macro. (DOC) [file pone.0079879.s012.doc]

**Confocal Microscope performance check acquisition protocols**

The acquisition protocols were developed and tested with the following instruments:

| **System (laser configuration)** | **Acquisition Software** |
| --- | --- |
| Leica SP1  (405nm/Argon/561nm/633nm lasers) | LCS 2.0 |
| Leica SP2  (Argon/561nm/633nm lasers) | LCS 2.61 Build 1537 |
| Leica SP5/SP5II AOBS  (405/Argon/543/633nm lasers; 405/Argon/561/594/633nm lasers) | LAS AF 2.6.0.7266 |
| Zeiss LSM 510 Meta  (Argon/543/633nm lasers) | LSM software 3.0 SP2  (Windows 2000) |
| Zeiss LSM 780  (405/Argon/561/594/633nm lasers) | Zen 2011 (black edition, 64bit) 7.0.4.287 |
| Nikon A1R  (405/488/561/638nm lasers) | NIS Elements 4.0 4.00.04 |

Due to the many different configurations available for each of these systems the protocols should only be regarded as guidelines and will have to be adjusted for the locally available instruments.

Although there are two Leica specific tests (spectral photometer accuracy, Z-galvo stability) all other system checks are suitable for confocal systems from other suppliers. Potentially all systems with file formats compatible with the Bioformats plugin could be used, however there are numerous inconsistencies in the metadata and image retrieval via the Bioformats macro extensions currently limiting the use of ConfocalCheck to the systems listed above.

Plain composite TIFF images and stacks created on other platforms can also be used for analysis, the metadata are entered manually when prompted by the ConfocalCheck macro.

**System preparation:**

- The confocal microscope and the lasers are switched on at least 60 minutes prior to recording any test data.
- The argon laser power is adjusted for normal imaging conditions (these are only guidelines based on our own instruments !):
  - Leica SP5 confocal: Set to ~20% with the LAS AF software *Laser Power* slider;
  - Leica SP1/SP2: LEVEL dial set to the 9 o’clock position.
  - Zeiss 510: Set the *Output[%]* slider to 50% in the *Laser Control* dialog box, giving a tube current of ~6.3A.
- Objective lenses are inspected and cleaned beforehand to ensure optimum imaging conditions.
- Images are recorded with 8bit (256 grey levels) bit depth.

**Saving the test data sets:**

On the Leica systems a single experiment file (.lif/.lei) should be created for each objective lens as the analysis macro searches for key words to identify particular data sets eg “bead”, “psf” and does not distinguish between “bead” images obtained with different lenses.

The key words are all in lower-case !

For the Zeiss (.lsm/.czi), Nikon (.nd2) and TIFF (.tif) files separate folders should be created for each objective lens with each folder containing the relevant image files eg. “bead.lsm”, “psf.nd2”, “bead.tif”.

Folder and file names must not contain any space characters as that will cause problems with ImageJ/Fiji !

**Performance checks**

| **Lasers**  Laser power | **Page** |
| --- | --- |
|  | 3 |
| Laser stability | 7 |
| **Objective lenses**  Field illumination    Spectral registration/colocalisation  Point spread function  Axial resolution/colocalisation | 8  9  10  11 |
|  |  |
| **Other Mechanical components** |  |
| Z-galvo stability  XY scanning galvos  Spectrophotometer accuracy  XY motorised stage accuracy | 14  15  16  17 |

**Measuring laser power**

**Requirements:**

- 10× objective lens
- Laser power meter with sensor mounted directly on the microscope stage (it is useful to have the sensor mounted in a slide-sized holder).

**Procedure:**

- Switch on all lasers at least 60min before the measurements and adjust the argon laser power for normal imaging conditions.

The argon laser power is set to the maximum power only shortly prior to taking the measurements as that can reduce the lifetime of the laser!

Alternatively one can take the measurements at a lower power setting eg. 25% or 50% and interpolate the results or even measure at the normal imaging power, in particular if there are known issues with running the laser at high currents.

- Select the appropriate wavelength on the power meter.

Essentially one wants to scan continuously only at a single spot to avoid fluctuating meter readings that occur due to beam blanking when scanning images.

Where this cannot be achieved one could scan at the slowest scan rate, with the largest image size and at the highest zoom factor.

The following steps describe setting up the spot scanning on different confocal systems:

**Leica SP1/SP2 confocal:**

- On the SP2 change the beam expander to position “---“ !
- Press the Singe Scan button to capture an image.
- Select the appropriate dichroic mirror in *Beam Path Setting* (depending on local system configuration):

**On SP2:** **On SP1:**

458nm: 458/514 458nm: RSP465

476nm: RSP500 476nm: RSP500

488nm: 488/561 488nm: 488/561/633

514nm: 458/514 514nm: RSP525

561nm: 488/561 561nm: 488/561/633

633nm: 488/561/633 633nm: 488/561/633

- Set the AOTF slider for the particular laser line to the maximum.
- Click on the *Bleach* icon. The *Bleach* dialog box appears.
  - Move the mouse over the captured image and place the cross-hair cursor in the centre of the image. Press the left mouse button to mark the point position.
  - Set *Duration* to >= 1min.
  - Press *Define*. Press *Recall*.
- In the *Advanced Timelapse* dialog box:
- Tick the *Bleach* box in the *Lapse1* settings.
- Press *Define*.
- Press *Start* to initiate scanning at the bleach point position.
- Adjust the XY stage and the focus position until the maximum meter reading is obtained. This position is used for all other measurements as well.

**For the other laser lines:**

- Remove the old bleach point by pressing the *Remove* button in the *Bleach* dialog box.
- Select the appropriate dichroic and laser line. Set the AOTF slider to 100%.
- Create a new bleach point as described above.
- In the *Advanced timelapse* dialog box:

Click on *Define* to deselect and click again on *Define*.

Press *Start* for the next measurement.

**Leica SP5 AOBS confocal:**

- Set the AOTF slider for the particular laser line to the maximum.
- In the *Beam Path Settings* tick the *Bleach Point* box in the *ROI Scan* settings.
- The *Bleach Points* dialog box appears in the *Acquisition* settings.
- Set the *Duration* to 60 seconds.
- Click on the *Draw point* icon of the imaging window and then click on the centre of the window to mark the position of the bleach point.
- Press *Start* to initiate scanning at the bleach point position.
- Adjust the XY stage and the focus position until the maximum meter reading is obtained. This position is used for all other measurements as well.
- Repeat for each laser line.

**Zeiss LSM 510 Meta**

- Select the appropriate dichroic mirror in the *Beam* *Path* *and Channel assignment* menu (depending on local system configuration):

458nm: HFT 458/514

488nm: HFT 488

514nm: HFT 458/514

543nm: HFT UV/488/543/633

633nm: HFT UV/488/543/633

- In the *Scan Control* menu select *Spot*.
- Press the *Spot Select* button. A single frame will be scanned. The crossed lines mark the spot position, the default centre position is fine.
- Set the AOTF slider for the particular laser line to the maximum in the *Excitation* control window.
- Click on the *Time Series* icon.
- Set the *Start Series* and *Stop Series* settings to *Manual.*
- Set the *Number* slider to the maximum (for example 50000)
- Press *StartT* to initiate scanning at the defined point position*.*
- Adjust the XY stage and the focus position until the maximum meter reading is obtained. This position is used for all other measurements as well.
- Press *Stop* to finish scan and repeat for each laser line.

**Zeiss LSM 780 confocal**

- Select the appropriate dichroic mirror in the *Light Path* menu (depending on local system configuration):

405nm: MBS 405

458nm: MBS 458/514

488nm: MBS 488

514nm: MBS 458/514

543nm: MBS 488/543

594nm: MBS 488/594

633nm: MBS 488/543/633

- In the *Acquisition Mode* menu change the *Scan Mode* to *Spot*.
- Press the *Spot Select* button. A single frame will be scanned. The crossed lines mark the spot position, the default centre position is fine.
- In the *Multidimensional Tools* Selection Panel tick the *Time Series* check box.
- In the *Time Series* dialog box set *Cycles* to 100000 and the *Start* and *End* options to *Mode: Manual*.
- Set the AOTF slider for the particular laser line to the maximum in the *Channels* control window.
- Press *Start Experiment* to initiate scanning at the defined point position*.*
- Adjust the XY stage and the focus position until the maximum meter reading is obtained. This position is used for all other measurements as well.
- Repeat for each laser line.

**Measuring laser stability using the transmitter light detector**

**Requirements:**

- 10× objective lens
- Slide to setup Köhler illumination

**Procedure:**

- Switch on all lasers at least 60min before the measurements and adjust the argon laser power for normal imaging.
- Setup Köhler illumination with a sample slide as the measurements are carried out using the transmitted light detector, then remove slide.
- Select the appropriate dichroic mirror or beam splitter:
  - Leica SP1: substr.
  - Leica SP2: RT30/70 beam splitter.
  - No selection required on the Leica SP5 AOBS.
  - Zeiss LSM 780: MBS T80/R20
  - Zeiss LSM 510: MBS NT80/20
  - Nikon A1: BS20/80
- Select the time lapse scan mode with the default scan settings but 256×256 pixel image size to reduce file size.
- Select the transmitted light PMT as detector (Zeiss: *T-PMT* or *ChD*; Leica: *PMT trans.*, Nikon: TD).
- Zoom in to achieve an even intensity distribution across the image, eg. 8× zoom or set to maximum.
- Set a common transmitted light PMT gain and offset for all laser lines, and adjust intensity for each line only with the AOTF to achieve an intermediate grey level of 120-150 (8bit). Use low PMT gain to minimise detector noise.
- Perform a time-lapse recording at 20s intervals with sequential scanning/multi-tracking (one laser at a time) for a minimum of 2 hours or longer if required. On a five laser system for example use the 405/488/561/594/633nm lines.

(On a Nikon A1 system only the 488nm line might be available for transmitted light imaging and only in conjunction with recording a fluorescence image. The analysis macro has been adapted for this.)

- Rename/save the image series as “laser” for the automated processing by the analysis macro.

**Measuring Field Illumination**

**Requirements:**

- Fluorescent plastic slides:

Chroma slides: blue slide for 405nm, green slide for 488nm

red AppliedPrecision slide for the 543/561/594/633nm laser lines or the equivalent Chroma slides

A No.1.5 coverslip can be mounted with a small drop of immersion oil to avoid scratching.

**Procedure:**

- Switch on all lasers at least 60min before the measurements and adjust the argon laser power for normal imaging.
- Visually inspect lenses to be tested and clean with abs. ethanol (or as specified by manufacturer) and Whatman 105 lens tissue as required.
- Mount fluorescent slide and scan with the appropriate laser line, dichroic and filters until you encounter the brightest signal (at the slide surface).
- Focus another 30-75 µm into the slide:

10x: 75 µm

20x: 50 µm

40x: 40 µm

63x/100x: 30 µm

- Set Zoom factor to 1 to obtain maximum field size.
- 512×512 pixel image size.
- Set Pinhole size to 1× Airy unit.
- Use low laser power to avoid bleaching of the slide, adjust gain/offset to optimise the dynamic range.
- 4× line/frame averaging might be necessary to reduce noise for image acquisition due to high gain settings.
- Record an image and rename according to the excitation wavelength eg. “field405”, “field488”, “field561”, “field633” etc.

(the wavelengths have to match the laser lines defined in the system configuration file !).

- Repeat for all objectives and lasers.

**Colocalisation/Spectral registration with Tetraspeck beads**

**Requirements:**

- Slide with 1µm Tetraspeck beads

**Procedure:**

- Switch on all lasers at least 60min before the measurements and adjust the argon laser power for normal imaging conditions.
- Visually inspect lenses to be tested and clean with abs. ethanol (or as specified by manufacturer) and Whatman 105 lens tissue as required.
- Mount bead slide and find isolated beads using low intensity epi-fluorescence to reduce bleaching. Always use the beads closest to the coverslip.
  - Set pinhole diameter to 1 Airy unit.
  - Set pixelsize to 256×256 pixels.
  - Set XY resolution to ~0.03µm (high zoom factor).
  - Use low laser power to avoid bleaching, adjust PMT gain and offset to optimise dynamic range. Use averaging if required (eg. 2× line averaging).
- Use the XYZ scan mode with appropriate Z stepsize and sequential scanning for 3-4 wavelengths to record a Z stack:

Example for Leica SP5 AOBS:

| Excitation [nm] | Emission range [nm] |
| --- | --- |
| 405 | 420 - 480 |
| 488 | 500 - 550 |
| 561 | 570 - 620 |
| 633 | 642 - 700 |

Example for Zeiss LSM 780:

Dichroic mirrors: MBS: MBS 488/543/633; MBS_InVis: MBS-405

| Excitation [nm] | Emission range [nm] |
| --- | --- |
| 405 | 410 - 495 (track 1 Ch1) |
| 488 | 508 - 550 (track 2 ChS1) |
| 543 | 580 - 620 (track 3 ChS2) |
| 633 | 637 - 747 (track 4 Ch2) |

| Objective magnification | Z-Step size [µm] |
| --- | --- |
| 10x | 1.0 |
| 20x | 0.25 |
| 40x-100x | 0.15 |

- - Rename/save the image series as “bead” for the automated analysis.

**Point Spread Function with PS-speck beads**

**Requirements:**

- Slide with green 175nm PS-speck beads

**Procedure:**

- Switch on all lasers at least 60min before the measurements and adjust the argon laser power for normal imaging.
- Visually inspect lenses to be tested and clean with abs. ethanol (or as specified by manufacturer) and Whatman 105 lens tissue as required.
- Mount bead slide and find isolated beads using low intensity epi-fluorescence to reduce bleaching. Always use the beads closest to the coverslip.
  - Set pinhole diameter to 1 Airy unit.
  - Set pixelsize to 256×256 pixels.
  - Set XY resolution to ~0.03µm (high zoom factor).
  - Reduce laser power to avoid bleaching, adjust PMT gain and offset to optimise dynamic range.
  - Use averaging if required (eg. 2× line averaging).
- Use the XYZ scan mode with appropriate Z stepsize and record a Z-stack (488nm excitation, emission 500-550nm):

| Objective magnification | Z-Step size [µm] |
| --- | --- |
| 10x | 0.5 |
| 20x | 0.25 |
| 40x-100x | 0.15 |

- - Rename/save the image series as “psf” for the automated analysis.

**Measuring Axial Spectral Registration**

**Requirements:**

- Mirror slide

**General Procedure:**

- Switch on all lasers at least 60min before the measurements and adjust the argon laser power for normal imaging conditions.
- Mount mirror slide.
- Set pinhole diameter to 1 Airy unit (or to the minimum diameter when trying to measure the maximum Z-resolution).
- 512×512 pixel image size; default scan parameters.
- Set up the reflection imaging mode:

Dichroic/Beam splitter:

- - Leica SP5 AOBS: set AOBS to reflection mode.
  - Leica SP1: substr.
  - Leica SP2: 30/70
  - Zeiss LSM 780: MBS T80/R20
  - Zeiss LSM 510: MBS NT80/20
  - Nikon A1: BS20/80
  - Create 10nm to 20nm wide detection windows centered around the excitation wavelengths eg. 483-493nm for the 488nm laser line.
  - Ideally one would like to perform the reflection scan simultaneously for all required laser lines eg. 405/488/561/633nm but that depends on the system configuration (see below).
- Focus on the mirror surface (brightest intensity and mirror scratches visible).
- Set the PMT gain and offset to maximise the dynamic range for each channel.
- Switch to the XZ scanning mode (line scan in X direction/changing Z position).
- Record the XZ-Scan/Stack simultaneously with 3,4 or 5 channels (depending on laser and detector configuration):

- - On a three channel system two scans might be required, 405/488/561 and 488/561/633, rename the first scan “axial405” and the 2^nd^ scan “axial488”.
  - On a 4/5 channel system with a 405nm laser rename “axial405”.

Whether you use the “axial405” or “axial488” keyword is not critical, it simply has to match the corresponding settings in the system configuration file.

- Repeat for each objective lens.

As the XZ reflection scans are performed quite differently on the various systems below more detailed instructions:

**Leica SP confocal systems with Z-galvo:**

- Setting up the reflection mode:

Choose the appropriate Dichroic/Beam splitter:

- - Leica SP5 AOBS: set AOBS to reflection mode.
  - Leica SP1: substr.
  - Leica SP2: 30/70

Using the spectral sliders for each PMT create 10nm wide detection windows centered around the excitation wavelengths eg. 483-493nm for the 488nm laser line.

- Focus on the mirror surface.
- Change the scan mode to XZY.
- Adjust the zoom settings to achieve a XZ resolution of 0.015-0.03µm/pixel (the x and the z pixelsize change simultaneously).
- Use the microscope focus drive to centre the XZ reflection bands in the middle of image.
- Set the PMT gain and offset to maximise the dynamic range for each channel.
- Record a single XZ scan and rename “axial405” or “axial488”.

**Zeiss LSM 510 Meta**

- Setting up the reflection mode:

Choose the appropriate Dichroic/Beam splitter:

- - NT80/R20

Adjust about 20nm wide detection windows around the excitation wavelengths using the Meta detector, for example:

| Excitation [nm] | Emission range [nm] |
| --- | --- |
| 488 | 484 - 506 ChS1 |
| 543 | 527 - 559 ChS2 |
| 633 | 623 - 666 ChS3 |

- Focus on the mirror surface.
- Set the PMT gain and offset to maximise the dynamic range.
- In the *Scan Control* settings change the scan mode to *Line*.
- Select *512* pixel format.
- Click on *Line Sel*. A single XY frame will be scanned. The default centred line position is fine.
- Adjust the zoom to achieve a pixel size of 0.15µm.
- In the *Scan Control* window click on *Z Stack*. Click on *Mark First/Last* and set the *Mark First* and *Mark Last* Z positions for the scan.
- Set the Number of Slices to 512 and the *Interval[µm]* to 0.15µm.
- Press *Start* to record a Z-stack of 512 slices at 0.15µm intervals and save as “axial405.lsm” or “axial488.lsm”.

**Zeiss LSM 780 confocal**

- Setting up the reflection mode:

Choose the appropriate Dichroic/Beam splitter:

- - MBS T80/R20 for *Visible* *Light*.
  - MBS T80/R20 for *Invisible light* (405nm/UV)

Tick the *Reflection* box in the *Light Path* settings.

Adjust the spectral range (10-20nm) of the detectors around the excitation wavelengths, for example:

| Excitation [nm] | Emission range [nm] |
| --- | --- |
| 405 | 400 - 410 Ch1 |
| 488 | 482 - 508 ChS1 |
| 543 | 537 - 548 Ch2 |

| Excitation [nm] | Emission range [nm] |
| --- | --- |
| 488 | 483 - 494 Ch1 |
| 543 | 534 - 560 ChS1 |
| 633 | 627 - 638 Ch2 |

- Focus on the mirror surface.
- Set the PMT gain and offset to maximise the dynamic range for each channel.
- Change the *Scan Mode* to *Line* in the *Acquisition Mode* settings.
- Adjust zoom to achieve a pixel size of 0.15µm.
- Record a Z-stack of 512 slices at 0.15µm intervals and save as “axial405.lsm” or “axial488.lsm” as the “.czi” XZ scans are not opened properly with the bioformats plugin.

**Nikon A1 confocal**

- Setting up the reflection mode:

Choose the appropriate Dichroic/Beam splitter:

- - BS20/R80

Adjust the spectral range of the *Spectral Free Band* detector, for example:

| Excitation [nm] | Emission range [nm] |
| --- | --- |
| 405 | 400 - 424 Ch1 |
| 488 | 478 - 508 Ch2 |
| 543 | 550 - 574 Ch3 |

- Focus on the mirror surface.
- Set the PMT gain and offset to maximise the dynamic range for each channel.
- In the *Scan Area* controls select the line symbol.
- Record a Z-stack of 513 slices at 0.2µm intervals (it was not possible to acquire 512 lines on the system we tested).
- Save as “axial405” or “axial488”.

**Measuring Z-Galvo stability**

(only on Leica confocal systems with Z-galvo)

**Requirements:**

- Mirror slide
- 40×, 63× or 100× oil immersion lens

**Procedure:**

- Switch on all lasers at least 60min before the measurements and adjust the argon laser power for normal imaging.
- Mount mirror slide.
- Set pinhole diameter to 1 Airy units (or to the minimum diameter when trying to measure the maximum Z-resolution).
- 256×256 pixel image size.
- Set up the reflection imaging mode with the 488nm laser line:
  - Leica SP5 AOBS: set AOBS to reflection mode.
  - Leica SP1: select dichroic substr.
  - Leica SP2: select dichroic 30/70.
  - Using the spectral sliders for the PMT create a 10nm wide detection range centered around the 488nm excitation wavelength (483-493nm).
- Focus on the mirror surface (brightest intensity and mirror scratches visible).
- Switch to the XZT scan mode. Adjust the zoom settings to achieve a XZ resolution of 0.015-0.03µm/pixel (the x and the z pixelsize change simultaneously).
- Use the microscope focus drive to centre the XZ reflection band in the middle of image.
- Set PMT gain and offset to maximise dynamic range.
- Record a time series (XYT scan mode) at 2s intervals for at least 1hour.
- Rename the image series as “zgalvo” for the automated analysis.

**Measuring the XY-scanning Galvos**

**Requirements:**

- Slide with reflective grid sample
- 10×objective lens

**Procedure:**

- Mount grid slide.
- Set pinhole diameter to 1 Airy unit.
- 1024×1024 pixel image size.
- Set up the reflection imaging mode with the 488nm laser line:

**Leica SP systems**

- - Leica SP5 AOBS: set AOBS to reflection mode.
  - Leica SP1: select substr. beam splitter.
  - Leica SP2: select 30/70 beam splitter.
  - Using the spectral sliders for the PMT create a 10nm wide detection range centered around the 488nm excitation wavelength (483-493nm).

**Zeiss LSM510 Meta**

- - Select the MBS NT80/20 beam splitter for the excitation laser and the two mirrors to direct the reflected light to the Ch2 detector.
  - Select the BP475-525 filter of Ch2 to detect the 488nm line.

**Zeiss LSM780**

- - Select MBS T80/R20 beam splitter.
  - Tick the *Reflection* box in *Light Path* settings.
  - Ch1 Detector range: 480-499nm.

**Nikon A1**

- - Select the BS80/20 main beam splitter and the 515LP secondary dichroic with the 482/35 emission filter for Channel1 (Standard Detector DU4).
- Focus on the square grid pattern. Rotate the slide manually or using the scan field rotation to align the grid squares parallel to the edges of the image.
- Record image with 4× line/frame averaging and 8× zoom.
- Rename/save the image as “grid” for the analysis macro.

**Measuring the spectral accuracy of the spectral detector on Leica SP confocal systems**

**Requirements:**

- Mirror slide
- 10× objective lens

**Procedure:**

- Switch on all lasers at least 60min before the measurements and adjust the argon laser power for normal imaging.
- Mount mirror slide.
- 256×256 pixel image size. Zoom factor 8× to obtain even intensity distribution.
- Open pinhole to the maximum.
- Set up the reflection imaging mode:
  - Leica SP5 AOBS: set AOBS to reflection mode.
  - Leica SP1: select substr. beam splitter.
  - Leica SP2: select 30/70 beam splitter.
- Create 5nm wide detection windows using the spectral sliders for the PMTs.
- Focus on the mirror surface (brightest intensity and mirror scratches visible).
- Adjust the PMT1 gain and offset to maximise the dynamic range for the detection window centred around the 488, 543 (or 561) and 633nm laser lines. Use the same gain and offset setting for all three wavelengths and adjust laser power with the AOTF to achieve similar peak reflection intensities. Use these same gain/offset settings for all the other PMTs.
- Perform a lamba scan (XYλ mode) for each PMT detector:
  - Leica SP1/2 settings: range 470-670nm in 2nm steps (100 steps)
  - Leica SP5 settings: range 470-668nm in 3nm steps (67 steps)
- Rename the image series as “scanpmt1”,“scanpmt2”, “scanpmt3” etc for the automated analysis.

**Measuring the motorised XY-stage performance**

**Requirements:**

- Slide with 1µm Tetraspeck beads
- 20× air objective lens

**Procedure:**

- Switch on lasers at least 60min before the measurements. We used the 633nm laser line for excitation (emission range 645-800nm) to minimise bleaching even when scanning over extended periods of time.
- **CAUTION !** The motorised stage needs to be initialised so ensure the objective lenses and the condenser are moved out of the movement path of the stage to avoid any damage in particular to the objective lenses!
- Set pinhole diameter to 1 airy units.
- 256×256 pixel image size. Pixelsize: 0.1µm/pixel.
- Mount bead slide and find beads.

There are two different protocols to assess either stage repeatability or accuracy.

For the repeatability test three beads several millimetres apart on the slide are marked and repeatedly imaged. To test accuracy the same bead is imaged repeatedly after the stage is moved in defined 10µm steps.

**Stage repeatability**

- Define the positions of three different beads (5-10mm apart). We only measured the repeatability over this very small range, the dimensions of a coverslip, but it could be extended to measure over the whole travel range with a suitable sample plate.

**Leica LAS AF**: Use the *Mark and Find* module to mark the positions.

**Zeiss LSM780 ZEN**: In the *Stage* acquisition parameter window click on *Mark* to define the positions.

**Nikon A1 NIS:** Define positions in the *XY* tab of the *ND Acquisition* window.

- Set up a time lapse recording to visit these positions 100 times.
- The image sequences for the different positions are labelled “stage1”, “stage2” and “stage3” in the Leica .lif/.lei experiment files and the TIFF format and “stage” in the other file formats.

**Stage accuracy**

- Define the position of a single bead (see system details above).
- Shift the position of the same bead within the field of view by a given value eg. 10 µm in X-direction. This can usually be done directly in the software. Then define this as the 2^nd^ position.
- Shift the bead in the other dimension by a given value eg 10 µm in the Y-direction and define this as the 3^rd^ position.
- Set up a time lapse recording to visit these positions 100 times.
- The image sequences for the different positions are renamed “stageacc1”, “stageacc2” and “stageacc3” in the Leica .lif/.lei experiment files and the TIFF format and “stageacc” in the other file formats.

**Measuring focus drift using the bead slide**

The stability of the microscope stage in Z-direction can also be assessed by imaging a single 1µm bead at only one position.

- - Use low laser power to avoid bleaching of the bead and record a time lapse sequence for several hours or overnight with suitable time intervals (for example 20s or 1min).
  - The image sequence is labelled “stage1” in the Leica .lif/.lei experiment files and the TIFF format and “stage” in the other file formats.
  - ConfocalCheck measures the bead intensity over time as a measure of focus drift.
