# Supplementary material for: ConfocalCheck - A Software Tool for the Automated Monitoring of Confocal Microscope Performance
Source: PLoS One. 2013 Nov 5;8(11):e79879. doi: 10.1371/journal.pone.0079879 (PMC3818239; doi:10.1371/journal.pone.0079879)
Supplement: Protocol S2 — Editing the System Configuration. Instructions on how to edit the system configuration file required by the ConfocalCheck macro. The configuration file "ConfocalCheck_Configuration.txt" contains information about the objective lenses and the laser lines used for the various assays. (DOCX) [file pone.0079879.s013.docx]

**Editing the ConfocalCheck system configuration file**

The configuration file only contains basic information on the laser lines used for the various performance tests as well as a list of the objective lenses required for setting up the HTML website files and when the objective type cannot be retrieved from the metadata.

The configuration file is called *ConfocalCheck_Configuration.txt* and has to be saved in the *ImageJ\macros* or *Fiji.app\macros* folder. The ConfocalCheck will display an error message if the configuration file is missing.

The supplied sample configuration file (S5) is based on the systems we have tested ourselves and works for the supplementary test data sets (S6-S8).

Before using ConfocalCheck with your own data you should edit the supplied configuration with a simple text editor (for example Microsoft Notepad/Wordpad or the ImageJ macro editor) as described below.

The syntax for each parameter is very simple, a keyword (eg. *systemname*) in lower case followed by colon (:), followed by one or more values separated by comma (,). Once all the parameters have been added in a line press RETURN/ENTER to create a line break.

Comments can be added above or below the parameter lines but avoid using the keywords.

Commas are only used to separate the items of a list. Press ENTER or RETURN after the last value to create a line break.

The colours are used for various graphs, the choices are:

black, blue, cyan, darkGray, gray, green, lightGray, magenta, orange, pink, red, yellow.

systemname: Leica_SP1

list of all lasers available on the system - when modifying the laser lines the corresponding lasercolour list needs to be amended as well

laserlines: 405,488,561,633

lasercolour: magenta,blue,green,red

objectives: 10x/0.30NA dry HC PL FLUOTAR (11506505),40x/1.00NA oil,63x/1.32NA oil,100x/1.35NA oil,100x/1.40NA oil

Enter the numerical aperture (NA) with two decimals as shown, followed by **NA**: 1.00 NA instead of 1.0NA; this is required to properly distinguish between lenses and for setting up the HTML files.

There should be no spaces between the comma and the objective magnification.

The objectives have to be listed in a single line (the line break here was introduced intentionally to show all the objectives).

In **laserlines** you specify the main system lasers, for a multi-line Argon laser only use the 488nm line. This information is used for the laser stability and field illumination tests

list of laserlines used for 1um bead colocalisation analysis

colocalisation: 405,488,561,633

list of laserlines used for axial405 resolution test

axial405: 405,488,561

list of laserlines used for axial488 resolution test

axial488: 488,561,633

laserline used for psf resolution test

psflaser: 488

list of laserlines used for lambda scan test of Leica SP microscopes

lambdascan: 488,561,633

systemname: Leica_SP2

list of all lasers available on the system - when modifying the laser lines the corresponding lasercolour list needs to be amended as well

laserlines: 488,561,633

Any parameter lines that are not used or not relevant, for example the axial405 line here, can be removed

lasercolour: blue,green,red

objectives: 10x/0.30NA dry HC PL FLUOTAR (11506505),100x/1.40NA oil

list of laserlines used for 1um bead colocalisation analysis

colocalisation: 488,561,633

list of laserlines used for axial488 resolution test

axial488: 488,561,633

laserline used for psf resolution test

psflaser: 488

list of laserlines used for lambda scan test of Leica SP microscopes

lambdascan: 488,561,633

systemname: Leica_SP5

list of all lasers available on the system - when modifying the laser lines the corresponding lasercolour list needs to be amended as well

laserlines: 405,488,561,594,633

lasercolour: magenta,blue,green,orange,red

objectives: 10x/0.40NA dry HCX PL APO CS(11506285),20x/0.70NA dry HC PL APO CS (11506513),40x/1.25NA oil HCX PL APO CS (11506251),63x/1.40NA oil HCX PL APO lambda blue(11506192)

list of laserlines used for 1um bead colocalisation analysis

colocalisation: 405,488,561,633

list of laserlines used for axial405 resolution test

axial405: 405,488,561

list of laserlines used for axial488 resolution test

axial488: 488,561,633

laserline used for psf resolution test

psflaser: 488

list of laserlines used for lambda scan test of Leica SP microscopes

lambdascan: 488,561,633

systemname: Leica_SP5II

list of all lasers available on the system - when modifying the laser lines the corresponding lasercolour list needs to be amended as well

laserlines: 405,488,561,594,633

lasercolour: magenta,blue,green,orange,red

objectives: 10x/0.40NA dry HCX PL APO CS(11506285),20x/0.70NA dry HC PL APO CS (11506513),40x/1.25NA oil HCX PL APO CS (11506251),63x/1.40NA oil HCX PL APO lambda blue(11506192)

list of laserlines used for 1um bead colocalisation analysis

colocalisation: 405,488,561,633

list of laserlines used for axial405 resolution test

axial405: 405,488,561,594,633

laserline used for psf resolution test

psflaser: 488

list of laserlines used for lambda scan test of Leica SP microscopes

lambdascan: 488,561,633

systemname: Leica_SP5III

list of all lasers available on the system - when modifying the laser lines the corresponding lasercolour list needs to be amended as well

laserlines: 405,488,543,633

lasercolour: magenta,blue,green,red

objectives: 10x/0.30NA dry HC PL FLUOTAR (11506505),20x/0.50NA dry HCX PL FLUOTAR (11506503),20x/0.70NA IMM/CORR HC PL APO CS(11506178),40x/0.85NA dry HCX PL APO CS Corr (11506295),40x/1.25NA oil HCX PL APO CS (11506251),63x/1.40NA oil HCX PL APO lambda blue(11506192)

list of laserlines used for 1um bead colocalisation analysis

colocalisation: 405,488,543,633

list of laserlines used for axial405 resolution test

axial405: 405,488,543

list of laserlines used for axial488 resolution test

axial488: 488,543,633

laserline used for psf resolution test

psflaser: 488

list of laserlines used for lambda scan test of Leica SP microscopes

lambdascan: 488,543,633

systemname: Zeiss_510_Meta

list of all lasers available on the system - when modifying the laser lines the corresponding lasercolour list needs to be amended as well

laserlines: 488,543,633

lasercolour: blue,green,red

objectives: 10x/0.30NA dry Plan-Neofluar,20x/0.50NA dry Plan-Neofluar,40x/1.30NA oil Plan-Neofluar,63x/1.40NA oil Plan-ApoChromat,63x/1.20NA W C-Apochromat

list of laserlines used for 1um bead colocalisation analysis

colocalisation: 488,543,633

list of laserlines used for axial488 resolution test

axial488: 488,543,633

laserline used for psf resolution test

psflaser: 488

systemname: Zeiss_LSM780

laserlines: 405,488,543,594,633

lasercolour: magenta,blue,green,orange,red

objectives: 10x/0.30NA EC Plan-Neofluar,20x/0.80NA Plan-Apochromat,63x/1.40NA oil Plan-Apochromat

colocalisation: 405,488,543,633

psflaser: 488

systemname: Nikon_A1

laserlines: 405,488,561,638

lasercolour: magenta,blue,green,red

objectives: 10x/0.30NA Plan Fluor Ph1 DLL,20x/0.45NA SPlan Fluor ELWD,60x/1.40NA Apo oil DIC N2

colocalisation: 405,488,561,638

axial405: 405,488,561

axial488: 488,561,638

psflaser: 488

systemname: Deltavision_Core

objectives: 10x/0.40NA UplSAPO,20x/0.50NA PH1 PlanFluorite UPLFLN,20x/0.70NA UPlanApo,40x/1.35NA oil Uapo/340,60x/1.40NA oil PlanApo,100x/1.35NA oil UPlanApo

As we only tested the stage performance of the Deltavision microscope, only the objective lens information was kept. All other parameters were deleted.
