# Supplementary material for: ConfocalCheck - A Software Tool for the Automated Monitoring of Confocal Microscope Performance
Source: PLoS One. 2013 Nov 5;8(11):e79879. doi: 10.1371/journal.pone.0079879 (PMC3818239; doi:10.1371/journal.pone.0079879)
Supplement: Protocol S3 — Using ConfocalCheck. Step-by-step instructions describing the use of the ConfocalCheck macro, also containing details of all the output files created by the software. (DOCX) [file pone.0079879.s014.docx]

**Step-by-step instructions on how to run the ConfocalCheck macro.**

1. Open ImageJ or Fiji. Install the ConfocalCheck macro with the *ImageJ/Plugins/Macros/Install…* function which prompts the user to select the macro text file “Macro_S4_ConfocalCheck1.txt”.

1. Once installed click on the target pattern icon in the tool bar to run the macro. If there are any open images the macro will not be executed, close all images first.

1. Next select the appropriate microscope system from the menu list by ticking one of the boxes, then click on OK. This list depends on the user-specific configuration settings in the system configuration file which needs to be edited (see Protocol_S2). The list below is for demonstration purposes only. When using the supplementary test datasets (Dataset_S6 to S8) tick the “Leica SP5II confocal” option.


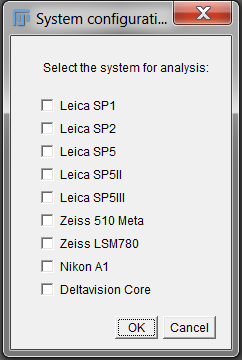


1. Select the file with the image data for a particular objective lens, for example in the Leica LIF or LEI file format as shown below. Then click Open.

(When using data recorded on other systems select one of the image files, for example bead.lsm, field488.czi or field488.nd2. When using TIF images select a correctly named TIF file here, eg. bead.tif or field488.tif).

The supplementary datasets Dataset_S6_LeicaSP5II_10x.zip

Dataset_S7_LeicaSP5II_20x.zip

Dataset_S8_LeicaSP5II_63x.zip are in the compressed ZIP file format and need to be extracted before use !

1. The objective details and the date of image acquisition are displayed in the Log window. All results are printed into the Log window which will automatically be saved at the end.

1. In the following “Options” menu you can select whether you want to create a new summary HTML website. If you tick this option you will be prompted to select the folder in which to save the HTML and JPEG files.

If you have created the HTML files previously and you want to update them with the new dataset only tick the box “Update existing summary website”.

If you tick the axial resolution box the macro will automatically create a vertical line across the centre of the images to obtain the line profiles when it encounters these images. Otherwise the user will be prompted to draw a vertical line, this would be the only user intervention required.

Some parameters cannot be retrieved from the metadata and have to be entered manually when prompted by the macro.

1. Depending on processing speed and the number of parameters to analyse the macro will run for about 30 to 60 seconds. At the end a message window will appear indicating which parameters have been analysed. The final window shows which html file was amended and the name of a backup file containing the previous data.

1. The main analysis report and the result TIFF images are saved in the same folder as the source data files.

The file name consists of the system name, the objective lens magnification and NA, the date of data collection and a description of its contents (more information on the following pages).

For example:

Leica_SP5II_10x_0.4_21-06-2010_measurements.txt

Leica_SP5II_10x_0.4_21-06-2010_AxialResolution_405nm.tif

Leica_SP5II_10x_0.4_21-06-2010_Lambdascan_result.tif

Leica_SP5II_10x_0.4_21-06-2010_Laserstability_488nm.tif

The corresponding main HTML file name in the website summary folder would be called:

Leica_SP5II_performance_checks.html

Opening this file in a web browser will provide easy access to many of the relevant instrument parameters recorded over time.


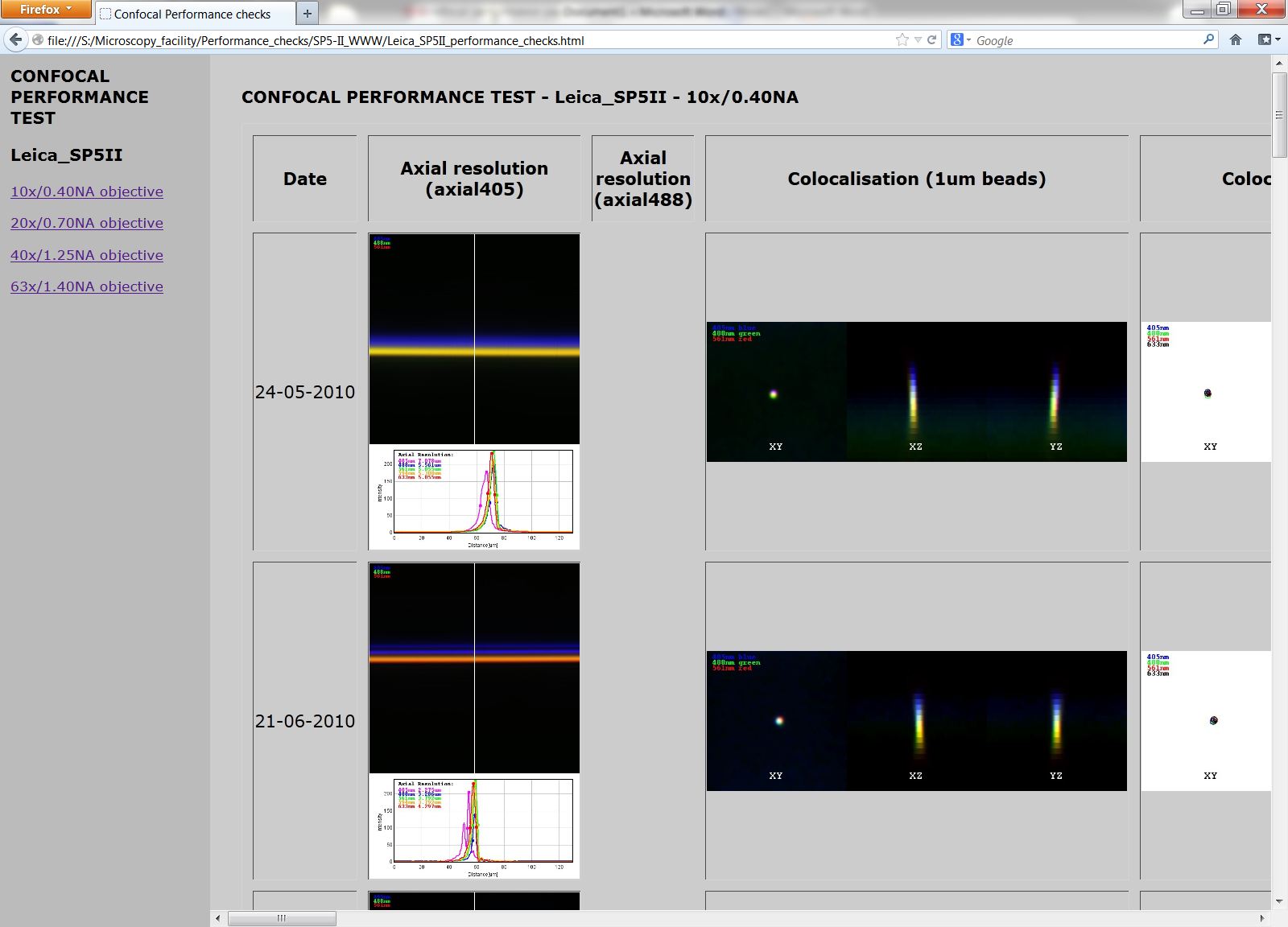


Select here the objective lens results you want to display in the main window on the right hand side.

**Description of the output files created by the ConfocalCheck Macro:**

All the files and measurements below are created when analyzing the provided test datasets (S6-S8) with the macro. The measurements are saved in the “measurements.txt” text files for each objective lens (for example: Leica_SP5II_10x_0.4_21-06-2010_measurements.txt). Additional HTML and JPEG files are created for the optional website summary.

| **Laser stability** | **Description** |
| --- | --- |
| Laserpower_data.txt | Tab delimited text file containing the average image intensity and the standard deviation for each image of the time series for each laser line. |
| Laserpower_Mean_Intensity.tif | Plot of the average image intensity over time, colour coded for each laser. Maximum intensity differences indicated as percentage. |
| Laserpower_Mean_Intensity_rescaled.tif | Same as above but rescaled intensity scale to show small intensity variations. |
| Laserpower_StdDev_Intensity_rescaled.tif | Plot of the image standard deviation over time, colour coded for each laser. Could be used as a measure for laser noise. |
| Laserpower_LineScan_Intensity_rescaled.tif | Horizontal intensity profile across the centre of the whole image of the first time point of the image series. Colour coded for each laser. Indicating pixel to pixel variation mainly due to laser noise. |
| Laserstability_[wavelength]nm.tif | Histograms showing percentage differences in pixel or image intensity over different timescales as indicated (µseconds, milli-seconds, seconds etc). Created for each laser line. |
| *Laser stability measurements:*  Minimum Intensity  Maximum Intensity  Mean Intensity  Standard Deviation  dIntensity [%]  *Laser noise in single horizontal line scan:*  *Mean Intensity*  *StdDev Intensity*  *CV [%]* | For each laser line the minimum/maximum/mean intensities and the standard deviation are calculated.  “dIntensity[%]” is the maximum intensity change expressed as a percentage.  Measurement of noise based on the intensity profile of a single horizontal line scan (see corresponding TIF image above). Calculation of mean and standard deviation as well as the CV value for each laser. |
| **Axial resolution and colocalisation** | **Description** |
| AxialResolution_405nm.tif and/or  AxialResolution_488nm.tif  *Axial resolution measurements:*  Axial resolution  Peak position | Top part: RGB overlay showing the reflection band of the first three laser lines (XZ scan).  Bottom part: Intensity profile along the white line for all the laser lines.The dots on the graphs mark the peak and halfmax positions used to calculate the FWHM/axial resolution as indicated.  Based on the FWHM the axial resolution is determined as well as the position of the reflection peak. This can be useful for the characterization of the axial chromatic correction for differents wavelengths. |
| **Field illumination** | **Description** |
| field_[wavelength]_result.tif | Contrast enhanced image of a fluorescence plastic slide. The black lines indicate the positions of the intensity profiles plotted on the right hand side with two different intensity scales to show small differences. Maximum intensity differences along the profiles are indicated as percentages. |

| **Field illumination** | **Description** |
| --- | --- |
| *Measurement field illumination:*  Minimum Intensity X Profile  Maximum Intensity X Profile  dIntensity X [%]  Minimum Intensity Y Profile  Maximum Intensity Y Profile  dIntensity Y [%] | Minimum and maximum intensities of the line profiles in X and Y direction as well as the maximum intensity difference as percentage. |
| **Bead Colocalisation** | **Description** |
| 1umBead_ori.tif | Different views of the Z-stack recorded with 1µm Tetraspeck beads; RGB overlay of the first three laser lines as indicated. |
| 1umBead_result.tif  *Colocalisation in 1um beads:*  Centroid X [um]  Centroid Y [um]  Centroid Z [um]  XY distance  Z distance | The same views as above showing the bead outline following segmentation with the centroid positions indicated.  The segmented image is used to calculate the 3D centroid for each wavelength.  The differences in the centroid positions for all possible combinations of two wavelengths are also calculated in XY and in Z which can be used to characterize the chromatic correction of the objective lenses. |
| **Point spread function (PSF)** | **Description** |
| PSF_projection_ori.tif | Different views of the Z-stack recorded with 175nm PS-speck beads at the calculated centroid position. |
| PSF_projection_result.tif | The same views as above using a pseudo-colour LUT to emphasize weak signals. |
| PSF_FWHM.tif  *Resolution measurements using PSF:*  FWHM axial  FWHM lateral | Line profiles obtained at the bead centroid position in XY and Z direction to measure the resolution as FWHM as indicated. Shown are the actual measurements (grey dots) and the fitted gaussian curve (black line).  Resolution measurements as obtained above, in Z and and XY direction. |
| PSF_Montage_ori.tif | Montage of the individual Z-stack images to analyse the PSF shape. |
| PSF_Montage_result.tif | Same as above with a pseudo-colour LUT to emphasize weak signals. |
| **Lambda scan/Spectrophotometer** | **Description** |
| Lambdascan_result.tif  *Lambda scan measurements:*  PMT1:  488nm line peak position [nm]  488nm line peak intensity  488nm line FWHM [nm]  PMT1:  561nm line peak position [nm]  561nm line peak intensity  561nm line FWHM [nm]  etc etc | Graph plotting the intensity of reflected laser light detected through a 5nm window when scanned between 470 and 670nm. Performed for each detector (colour coded) at three laser lines (indicated by three thicker grey lines).  For each of the detectors/PMTs there are three sets of measurements corresponding to the laser lines used for the scan.  The “peak position” indicates the wavelength at which the maximum reflection occurs, which should be close to the wavelength of the laser. The corresponding peak intensity is also saved. The FWHM of the peaks can be an indicator of potential issues but it is easier to examine the shape of the graphs in the TIF image. |
| **XY galvos** | **Description** |
| grid_result.tif | Contrast enhanced image of the square grid used to assess image distortions caused by the scanning galvos. |
| **XY stage** | **Description** |
| Stageposition1_XYplot.tif | A number of files are created for each bead for the stage repeatability analysis:  Plot of the XY bead centroids during the time course. The red dot marks the average position, the red rectangle the standard deviation calculated separately for the X and Y coordinates. |
| Stageposition1.tif | Multi-page TIFF stack with the masked bead image sequence. |
| Stageposition1_data.txt  *Measurement XY stage repeatability*:  Stage position 1: X [mm]  Stage position 1: Y [mm]  Bead position 1: Repeatability X mean [um ]  Bead position 1: Repeatability X StdDev  Bead position 1: Repeatability Y mean [um ]  Bead position 1: Repeatability Y StdDev | Tab delimited text file containing the bead centroid XY coordinates as well as minimum/maximum/average bead intensities during the time course.  The intensity values can be useful to assess bleaching or to measure focus drift when not using the Z-galvo method.  Record of the actual motorized stage positions as retrieved from the metadata.  Average X and Y centroid positions for each of the beads, together with the standard deviation which could be used as a measure of how well the stage/bead returns to its programmed position. |
| Bead_intensity_result.tif | Intensity plot showing the average bead fluorescence intensity for all beads during the time course. |
| *Measurement XY stage accuracy:*  Distance between stage positions 1-2[um]  Average bead distance [um]  Average deviation from stage distance [um]  StageAccuracy_data.txt  StageAccuracy_Position1_XYplot.tif  StageAccuracy_Bead_intensity.tif  StageAccuracy_Bead_images.tif | A number of additional files and measurements are created for this very basic stage accuracy analysis:  Essentially we compare how much the stage has moved (according to the positions retrieved from the metadata) with the movement of the beads as determined from the centroids:  The average distance travelled by the bead between the different positions is determined (from pos.1 to pos. 2, from 2 to 3, from 3 back to 1). The “deviation” of this measured distance from the stage travel distance serves as marker for the accuracy of the movement.  Tab delimited text file containing the bead centroid XY coordinates, the average bead intensities during the time course and the distances travelled by the bead between the three different positions.  This is the same bead centroid plot as described above but using the accuracy dataset, where the same bead is imaged at different positions  Intensity plot showing the average fluorescence intensity for the bead at the different positions during the time course.  Multi-page TIFF stack showing the overlay of the bead at the different positions. |
| **Z Galvo stability** | **Description** |
| Zgalvo_result.tif  *Measurement Z Galvo stability:*  Minimum Z position [µm]  Maxmimum Z position [µm]  dPosition [µm]  Zgalvo_data.txt | Upper part: graph tracing the position of the peak reflection band during the time course (XZT scan mode). The orange line marks the average position.  Minimum/maximum Z position and the maximum difference (“dPosition”) are indicated. Temperature changes and air flow can have a major effect on this, rarely equipment fault in our hands.  Lower part: graph showing the corresponding width (FWHM) of the reflection band as large variations can sometimes reveal vibrations affecting the system.  Tab delimited text file containing the peak position of the reflection band and the corresponding FWHM for each time point. |
