# Supplementary material for: ConfocalCheck - A Software Tool for the Automated Monitoring of Confocal Microscope Performance
Source: PLoS One. 2013 Nov 5;8(11):e79879. doi: 10.1371/journal.pone.0079879 (PMC3818239; doi:10.1371/journal.pone.0079879)
Supplement: Text S1 — The supplementary text describes in detail the type of analysis that can be carried out when using ConfocalCheck with the standardised test data sets. (DOC) [file pone.0079879.s020.doc]

**The supplementary text describes in detail the type of analysis that can be carried out using ConfocalCheck and the standardised test data sets.**

**The examples presented here have been collected over several years in the MRC CSC microscopy facility. As most tests are not instrument specific this section should assist in identifying potential issues with the microscope and help with the detailed characterisation of individual components like the objective lenses.**

**Any system or component specific details mentioned are only used for illustration purposes, as the detailed analysis of systems from any vendor over a longer period of time would provide numerous examples of component failure or misalignment.**

The results have been grouped into three main sections as in the image acquisition protocol (S1):

| **Lasers**  Laser power - *Long-term changes in maximum laser power* | **Page** |
| --- | --- |
|  | 2 |
| Laser stability - *Fluctuations in laser power – from μs to hours* | 2 |
| **Objective lenses**  Field illumination    Colocalisation analysis with Tetraspeck beads  Comparing the chromatic correction of different objective lenses across different microscopes  Axial chromatic correction and axial resolution  The point spread function | 5  5  6  7  8 |
|  |  |
| **Other Mechanical components** |  |
| Spectrophotometer accuracy - *Spectral detector performance*  The XY scanning galvos  Z-galvo stability - *The Z-galvo, temperature and focus drift*  XY motorised stage accuracy *- Motorised stage performance* | 9  9  10  11 |

**Section 1 – Lasers**

**Long-term changes in maximum laser power**

We measured maximum laser power monthly as a simple way of monitoring the entire illumination light path, ranging from the lasers, the laser merge module and the AOTF (acousto-optical tunable filter) to the fibre optic light guides that deliver the laser light into the scanhead . Figure S1A shows the laser power of the 488nm line of the Argon laser on one of the confocal systems. The power dropped significantly from July to September of the first year partly due to alignment drift as subsequent service visits (black arrows) increased laser power again by about 60% simply be re-adjustment of the laser-fibre coupling. The following decline in output power was caused by a drop in the transmission of the optical fibre connecting the Argon laser with the merge module. The replacement of that light guide (marked ‘R’ in Fig. S1A) restored the 488nm laser power back to 4.7mW. The newly installed 633nm HeNe laser however showed very little power variation once properly aligned – the increase at the last data point is the result of the comprehensive laser realignment following the fibre replacement. The 633nm HeNe laser showed generally little power variation on our other confocal microscopes as well (data not shown) which can be attributed to its position in the merge module as the 633nm laser is directly launched into the output fibre connected to the scan head.

Substantial fluctuations in laser power were also observed in a newly installed confocal microscope (Fig. S1B), suggesting that all systems, new or old, should be monitored regularly. The decrease in Argon laser power from May to July could have resulted from a combination of failing laser and/or failing Argon laser fibre. The laser stopped working altogether (indicated by asterisk in Fig. S1B) but following Argon laser replacement output continued to drop. The Argon laser fibre was eventually replaced (‘R’) and power output rose five-fold. The output of the other visible lasers like the 561nm DPSS laser was unaffected.

While these variations in maximum laser power may not have affected the day-to-day operation as users would have adjusted laser power and detector gain to capture ‘good’ images, it would be best practice to measure and adjust laser power for all applications where laser intensity is critical e.g. comparative quantitative imaging, FRAP or FRET, in particular when experiments have to be carried out over many months.

**Fluctuations in laser power – from μs to hours**

To assess the temporal stability of the lasers during the course of a typical experiment we measured laser intensity with the transmitted light detector of the confocal system as a time lapse recording as had been suggested previously . In cases where we suspected potential issues we also recorded overnight. The transmitted light path was chosen as it provided the most stable output on our systems. Measuring laser intensity with a mirror slide in reflection mode was not useful as small fluctuations in room temperature caused focus variations that changed the reflected light intensity significantly even with the confocal pinhole fully opened (data not shown).

Figure S2A shows the laser stability of one of the confocal systems. The average image intensity was plotted for each laser line channel versus time, together with the room temperature. The laser power fluctuated between 4 – 10% for the visible lasers while the 405nm laser showed additional higher frequency variations of about 14%. Autocorrelation analysis of the time series showed that the period of the intensity changes was the same as that of the temperature fluctuations (~28 minutes). One explanation for this could be the temperature dependent polarisation of the fibre optics – connecting the output from the merge module with the confocal scanhead - which could modulate the intensity of the polarised laser light. The effect of room temperature can easily be assessed for example by changing the rate or direction of air flow or switching the air conditioning unit off temporarily.

However the internal cooling and heat dissipation mechanisms in the laser unit can have a much stronger effect on the laser intensity than room temperature alone as we discovered when switching off the Argon laser with its large cooling fan while acquiring a time-lapse series. As the system temperature rose by several degrees Celsius - measured with a temperature sensor mounted directly on the housing of the electronics/laser unit – the output from the remaining lasers dropped by 30-50% (data not shown). Generally it took 1.5 to 2 hours for laser power to stabilise, another factor to consider when performing quantitative imaging.

On another confocal system the effect of room temperature effect was less pronounced (Fig. S2B) probably due to different air ventilation systems (rate and direction of air flow, room size etc.). Here laser intensity varied only by 2.6 to 3.8% and showed higher frequency components not observed in the other system.

Apart from giving the user a feel for how the excitation light intensity can change during the course of an experiment and how environmental factors like airflow and temperature can affect the system we found these stability tests also helpful for testing the proper functioning of the lasers.

When noticing rapidly changing power meter readings from a 405nm diode laser (the same as shown in Fig. S2B), we performed a number of long term recordings which confirmed the abnormal behaviour (Fig. S2C). During the investigation into the cause of the erratic fluctuations most parts of the UV light path were replaced including the AOTF driver and the diode laser itself, always followed by long term time lapse recordings to test stability as the output was often stable while the service engineer was on site. Finally the AOTF module (containing the AOTF crystal and a temperature sensor) was replaced resulting in stable laser output.

We also analysed the short-term laser intensity fluctuations (noise) within individual images, on the time scale of μ-seconds to seconds . Noise contributions from the transmitted light PMT and the read-out electronics were minimised by using low PMT gain and high laser power. The PMT gain and offset was kept the same for all the laser lines. Spatial variation was reduced by using maximum zoom.

A simple line graph of the intensity along a single horizontal scan line shows the pixel-to-pixel intensity variations as the laser scans across the field typically with a pixel dwell time in the μ-second range (Fig. S3A). From the mean intensity and standard deviation the coefficient of variation (CV) was calculated . This can be used to compare the different lasers (the same as shown in Fig. S2B) but the CV values were all very low indicating little variation (405nm: 0.45%, 488nm: 0.64%, 561nm: 0.48%, 594nm: 0.49%, 633nm: 0.49%).

In addition we measured the intensity distribution of the entire image by calculating and plotting the standard deviation for all the images in the time series to assess whether the laser noise levels change over time (Fig. S3B). Again the intensity variations were very low except for the 594nm HeNe laser that showed a slightly more erratic behaviour which was however not apparent in subsequent performance checks (data not shown).

To create a more quantitative and easy to understand visual output for the laser intensity fluctuations on the different time scales we calculated the differences between subsequent intensity measurements as a percentage change . We then calculated the relative frequencies of the changes and plotted them in histograms. Figure S3C/D shows the analysis for the 488nm line of the Argon lasers shown in Fig. S2A/B. Starting with the calculated pixel dwell time of 9.8μs (Fig. S3C) obtained from the horizontal line scans the green bar indicates that in 40% of cases the intensity of adjacent pixels was identical (0% difference), 45% showed a 1% difference etc. The orange and red lines simply indicate the 5% and 10% difference marks. The 2.5ms data were obtained from measuring vertical intensity profiles in the images as with the 400Hz scan speed used it takes ~2.5ms for the laser to reach the same position in the next scan line.

The fluctuations between subsequent images were shown in the 20s histogram, as 20s was the time interval used for the time lapse recording. We also used 1min, 10min and 1 h intervals for our analysis to quantify intensity fluctuations over those periods. These were chosen somewhat arbitrarily but should reflect the duration of commonly used scan patterns. The comparison of the two argon lasers showed increased short term noise levels in one of them (Fig. S3D).

All these plots and measurements are automatically generated by the ConfocalCheck macro and might serve to highlight potential issues with the lasers or laser delivery systems. In case of increased laser noise for example, if that has an effect on image quality this could be counteracted by image averaging.

**Section 2: Characterisation of the objective lenses**

**Field illumination**

We used fluorescent plastic slides to characterise the spatial homogeneity of the illumination . This depends on the objective lenses themselves as well as other components in the excitation light path (laser alignment, UV/405nm corrective optics etc). The majority of problems we encountered were related to the alignment of the 405nm diode laser on our Leica SP microscopes, where either the laser-fibre alignment or the objective specific 405nm correction lenses had drifted. A typical example is shown in Fig. S4A, the brightest fluorescence intensity is in the upper right quadrant rather than in the centre of the field of view (as indicated by the crossing of the two black lines).

To spot any anomalies quickly the images were contrast enhanced by linear histogram stretching. Additionally we plotted the intensity profiles along the X and Y lines (black lines in Fig. S4A) on two different intensity scales as that proved useful in particular when assessing small intensity variations (Fig. S4B). As the objective lenses and test slides were cleaned before the performance checks any irregularities were due to other optical components in the light path. That way we eventually identified an oil contaminated tube lens on a SP2 confocal system (Fig. S4C).

It is also worth taking images with the transmitted light detector as immersion oil residue, finger prints and other contaminations on the front lens of the condenser will also show up, particularly obvious when rotating the lens while scanning (data not shown).

The dark band observed at the top of the 405nm field image (Fig. S4D, red arrows) was due to the slow opening of the 405nm laser shutter at the start of image acquisition. This problem was intermittent at first but got worse over several months and finally the shutter failed altogether.

**Colocalisation analysis with Tetraspeck beads**

Optimal alignment is a prerequisite for colocalisation studies where the spatial distributions of several fluorophores need to be measured very accurately. We checked the colocalisation by recording Z-stacks of fluorescent 1μm Tetraspeck beads and measuring the X,Y and Z coordinates of the bead centroids for each wavelength. Serious misalignments were easily spotted in the RGB overlay view as seen in Fig. S5A. Although there was only a small deviation of the blue signal (405nm excitation) in the XY view as indicated by the arrow, the blue signal didn’t even overlap with the images obtained with the other wavelengths in the Z-direction due to the wrong 405nm correction lens.

The outline view shows the outline of the segmented bead images used to calculate the bead centroids, indicating an otherwise very good spectral registration of this 40×/1.25NA Plan-Apo oil immersion lens. The small squares mark the centroid positions. As the overlay image was based on the three primary colours red, green, and blue, we plotted the 4^th^ channel (633nm excitation) in black as indicated.

The re-adjustment of the 405nm correction lenses was the most frequent alignment that had to be carried out by service engineers and involved overlaying the bead images obtained simultaneously with 405nm and 543/561nm excitation at high zoom settings.

The reason for the displacement of the bead images obtained with different wavelengths on another system (Fig. S5B, before and after alignment) remained elusive until it was discovered that the confocal scanhead was not properly attached to the microscope body.

**Comparing the chromatic correction of different objective lenses across different microscopes**

To examine the chromatic correction of different objective lenses in a more quantitative way we calculated the average centroid distances for the image pairs obtained with 488/561(or 543)nm and 488/633nm excitation. The XY and Z distances of all combinations of centroid pairs are directly calculated by ConfocalCheck. These measurements can be used not only to compare different lenses on the same system but across different confocal microscopes, in our case three Leica SP5 systems as described below.

The lateral XY centroid distances for the 488/561 pair for both the 40×/1.25NA oil HCX PL APO CS and the 63×/1.40NA oil HCX PL APO lambda blue objectives were around 50nm - well below the optical resolution limit -, with the 488/633 distances slightly longer (Fig. S5C). The axial (Z) centroid distances were however considerably longer for the 63×/1.40NA lambda blue objectives, probably reflecting the different chromatic correction of the 63×/1.40NA lambda blue lens which is supposed to have a better chromatic correction in the blue/green range than in the red/far-red part of the spectrum.

Although we typically only recorded one bead per objective for our performance tests to simplify image analysis, the plots averaged from data obtained over 6-12 months clearly show the differences between different types of objective lenses as well as the similarity of the two tested 40×/1.25NA and 63×/1.4NA lenses. This suggests that our approach is both sensitive and robust enough to perform these quantitative comparisons.

We also compared 20× objectives lenses from three different Leica SP5 microscopes using the same metrics (Fig. S5D). While the plan-apochromat (20×/0.70NA HC PL APO CS) and the Fluotar fluorite (20×/0.50NA HCX PL FLUOTAR) lenses were very well corrected for the green-red part of the spectrum as indicated by the small XY and Z centroid distances (488/543,488/561 pairs), the fluorite lens showed a large axial displacement of the far-red bead image (488/633 pair) of about 2μm. The fluorite lens would therefore not be suitable for colocalisation studies using dyes emitting in the red/far-red region of the spectrum but many confocal users would not necessarily be aware of that.

**Axial chromatic correction and axial resolution**

In order to examine the axial chromatic correction of the objective lenses, how well the lenses are able to focus light of different wavelengths into the same focal plane we measured the reflection of laser light from a mirror slide . This is straight forward to achieve on the Leica confocal microscopes using the fast XZ scanning mode with the Z-galvo, but can be setup on all other systems we tested as well.

Fig. S6A shows the overlay of the reflection bands from three XZ scans (488/543/633nm laser lines) with the corresponding line intensity plot below. The graph shows a good superposition of the reflection bands indicating good axial chromatic correction for this particular Plan-Apo objective (Leica 20×/0.70NA HCX PL APO CS multi-immersion). In contrast the red (633nm) reflection band is displaced by almost 2μm for the less well corrected fluorite lens (20×/0.50NA HCX PL FLUOTAR), similar to the results of the bead colocalisation analysis shown above for the same lens (Fig. S5D).

For a more detailed comparison of the axial chromatic correction of different lens types we examined the reflection bands for six visible laser lines ranging from 458 to 633nm. When plotting the distances of the peak intensity positions relative to the reflection peak of the first 458nm line one can clearly observe different patterns (Fig. S6C). The reflection peaks for the well corrected 10×/0.4NA Plan-Apochromatic lens were spread over a much narrower range compared to the 10×/0.3NA Fluotar lens. The graph suggests that for the 10×/0.3NA Fluotar both the blue (458nm) and the red (633nm) light are considerably displaced from the blue-green/orange part of the spectrum.

As the numerical aperture of the lenses increases the reflection peak distances decrease as can be seen for the 20×/0.70NA PL APO as well as for the oil immersion lenses tested (Fig. S6C/D). The conventional Plan-Apochromatic lenses (40×/100×) display very similar curves with the blue-green to red components of the spectrum (514-633nm) focused close together. In contrast the lambda blue 63×/1.4NA objective focusses light of 488 to 594nm to within about 100nm. The better correction in this part of the spectrum is presumably to the detriment of the far-red end of the spectrum beyond 633nm as shown above in the analysis of fluorescent beads (Fig. S5C), where noticeable bead displacement was observed in the far-red. The peak to peak distances of the various reflection bands were very similar for a particular type of objective lens across different Leica SP5 systems and showed generally very little variation over the course of 12 months (data not shown).

The FWHM (full width at half-maximum intensity) of the reflection band can also be used as a measure of the axial resolution of the system and to probe the optical sectioning capabilities of the confocal microscope . Fig. S6E shows the resolution measurements for the three oil immersion lenses on one of our confocal systems as a function of pinhole size. The axial resolution (as FWHM) and the reflection band peak positions used above are automatically measured during the image analysis with ConfocalCheck (Fig. S6A/B).

**The point spread function**

The point spread function (PSF) was recorded with sub-resolution 175nm beads (488nm excitation) and is another useful measure for characterising the imaging system and in particular the objective lenses . We used it to determine the axial and lateral resolution (Fig. S7B) and to create a visual output to easily spot any changes in the diffraction patterns potentially indicating lens damage (Fig. S7A/C). Having a recorded PSF is also useful for the subsequent deconvolution of confocal image stacks if required.

Although we found clear differences in the diffraction patterns when comparing the PSF of similar lenses from different microscopes side by side (data not shown) we didn’t observe any noticeable changes in the individual patterns of our objective lenses over the last 3 years.

The lateral and axial resolution measurements essentially remained constant exemplified here for the 63×/1.4NA Plan-Apo lenses available on the three Leica SP5 confocal microscopes (axial resolution: 500±42nm, 598±56nm, 565±69nm; lateral resolution: 253±14nm, 261±11nm, 258±20nm, data averaged over 9-15 months), despite relying on measuring just a single bead per session.

**Section 3 - Other Mechanical components**

**Spectral detector performance**

Besides the laser illumination and objective lenses we decided to monitor a range of other components relevant for confocal operation in particular in areas where we had encountered problems over the years. One component critical for the spectral separation of light emitted from fluorescent samples on the Leica SP confocal systems are the spectral slider units. They are mounted in front of the PMT detectors to select the appropriate wavelength range while reflecting the unwanted wavelengths towards other detectors. This is achieved by moving two small mirrors and any incorrect positioning of these mirrors could reduce or even block the wanted signal or shift the wavelength range which would not be obvious to the confocal user.

We tested the spectral sliders for each detector by performing a wavelength/lambda scan in reflection mode using 3 laser lines in the visible range (488/543 or 561/633nm). Fig. S8A shows these lambda scans, as the 5nm wide detection window is moved across the 200nm wavelength range the intensity of reflected light peaks at the specific laser lines. The arrow marks the curve for detector 5 which was barely detectable and the mirror slider unit was subsequently replaced. In Fig. S8B the mirror movement was restricted in the spectral slider unit for PMT1 therefore scanning with a much wider detection window indicated by the broader reflection bands. As our confocal checks were carried out monthly we could also follow the gradual changes in the spectral detection system over time on another system (Fig. S8C). After recalibration of the spectral sliders for the 633nm line by a service engineer full functionality was restored (Fig. S8C: ‘October’).

**The XY scanning galvos**

To evaluate the presence of distortions in the scanned images regular square grid patterns are used as this would be impossible to assess with biological samples. As careful examination of the images was usually sufficient to spot deviations from the square shape we didn’t analyse the images in more detail. Fig. S9A shows the image of the grid pattern at different zoom settings, with the squares clearly elongated in the Y-direction at the top of the image. This was resolved by recalibration of the Y-galvo settings. More subtle were the effects caused by the X-galvo on another system stretching the square grids in the X-direction on the right hand side of the image as indicated by the red squares (Fig. S9B).

**The Z-galvo, temperature and focus drift**

Rapid fine focus movement on the Leica confocal microscopes is usually carried out by the Z-galvo driven sample holder rather than the focus drive of the microscope (travel range 150µm on a Leica SP1/2 system, 500-1500µm on a Leica SP5). In particular for long scans or time lapse recordings stage stability is important. To monitor Z-drift we carried out XZ scans of a mirror slide in reflection mode over time . In Figure S10A we plotted the peak position of the reflection band, showing abnormally large Z-drift (> 1µm/hour) on one unit. As we noticed on some systems that the width of the reflected light band changed considerably from image to image (see inset Fig. S8B) in particular when the microscope is affected by vibrations we also measured the FWHM of the reflection band. Fig. S8B shows the FWHM plotted as a function of time with very little variation. This system was on an active anti-vibration table. Similar measurements on systems with passive tables in different locations displayed much larger fluctuations (data not shown). This approach might therefore help to assess room vibrations.

Performing timelapse XZ scans was also useful to monitor the effects of air flow and temperature changes on the focus stability of the microscopes . An example of a relatively stable system is shown in Fig. S10C. This confocal microscope had a large environmental chamber covering most of the microscope which was set to 37ºC to monitor focal drift at the temperature used for live cell imaging experiments. A temperature sensor was directly mounted on the metal surface of the Z-galvo stage. The stage temperature was slightly lower than the 37ºC air temperature and fluctuated periodically by around 0.1ºC, close to the resolution limit of the temperature probe (Fig. S10C, grey lines). Averaging the temperature reads over 60 seconds clearly showed periodic 30 minute fluctuations mirroring the independently measured room temperature changes (data not shown). These tiny temperature fluctuations were sufficient to cause periodic focus changes in the range of ~0.5µm as calculated from the position of the reflection band.

To confirm that these focus changes were not simply due to temperature effects on the Z-galvo stage alone we replaced it with a non-motorised manual stage and recorded the fluorescence of 1µm beads over time with a 40×/0.85NA air lens. Fluorescence intensity of the beads changed periodically by about 30% as the beads moved slightly in and out of focus over time with the temperature fluctuating again by about ~0.1 ºC (data not shown).

This analysis of time lapse recordings of fluorescent beads is also incorporated into ConfocalCheck. Simply record a single bead as detailed in the *XY motorised stage accuracy* section of the acquisition protocol (Protocol S1; except only use one bead, do not move the stage and record it as long as required, typically for several hours or overnight !). ConfocalCheck will measure and plot the bead intensity over time which can be useful when evaluating environmental factors like airflow and temperature. Combining this with room or stage temperature measurements might help to identify the factors causing focus drift .

An adjacent confocal system without environmental chamber was exposed to much higher air flow from the air conditioning units and we measured Z movement changes in the range of 6-9 µm over 30 minutes (data not shown) leaving the system only suitable for much shorter scans of fixed samples.

The effect of temperature should always be taken into account when experiencing drift problems. When switching on the microscopes the temperature of the motorised stages on our systems rose by about three degrees from around 21ºC room temperature to 24ºC which took about to 2 hours stabilise. It took about 4 hours for the stage temperature to stabilise when the heater of the environmental chamber was turned on, much longer than the air temperature as measured by the temperature probe of the heating unit.

**Motorised stage performance**

The final system component we tested was the motorised stage commonly available now on many research microscopes for multiple site time lapse recordings or for scanning large sample automatically. This was in response to observing drift on a Deltavision deconvolution microscope where the sample seemed to drift laterally during the course of live cell imaging experiments. Fig. S11A shows the movement of a fluorescent 1µm bead that was tracked over 2 hours with considerable movement in both X and Y direction. After stage replacement this problem was resolved (Fig. S11A inset).

To measure the performance of the motorised stages on our confocal microscopes in general we looked at the repeatability and accuracy of stage movement. Repeatability was assessed by marking the positions of three different 1µm beads spaced 5-10mm apart on our bead slide and moving to all 3 positions 100 times. In Fig. S11B we plotted the averaged bead centroid positions obtained from two test runs on two different systems. The standard deviations indicate that the stages move back to the same position within less than 0.5µm distance in Y direction and slightly worse in X-direction.

To assess the accuracy of stage movement - how far the stage actually moves when asked to move a certain distance - we first defined the starting position of a single bead within the field of view. Then we moved the stage by 10 µm in X direction according to the stage positioning controls in the software and defined this as the 2^nd^ position, the stage was then moved down 10 µm in Y direction (3^rd^ position). The beads were imaged again by visiting the three positions 100 times. ConfocalCheck calculates the differences between the measured bead centroid positions and the actual stage positions obtained from the metadata. On average the measured bead positions differed by about 10-20% (1 to 2µm) from the indicated stage positions (data not shown).

**References**

1. Hibbs AR, MacDonald G, Garsha K (2006) Practical Confocal Microscopy. In: J. P, editor. Handbook of Biological Confocal Microscopy. 3rd ed. New York: Springer. pp. 650-671.

2. Zucker RM (2006) Quality assessment of confocal microscopy slide-based systems: instability. Cytometry Part A : the journal of the International Society for Analytical Cytology 69: 677-690.

3. Zucker RM, Price O (2001) Evaluation of confocal microscopy system performance. Cytometry 44: 273-294.

4. Zucker RM (2006) Evaluation of confocal microscopy system performance in "Methods in molecular biology - Cell Imaging Techniques, Methods and Protocols"

Walker JM, editor. Totowa,: Humana Press Inc. 77-135 p.

5. Swedlow JR, K H, Andrews PD, Roos DS, Murray JM (2002) Measuring tubulin content in Toxoplasma gondii: a comparison of laser-scanning confocal and wide-field fluorescence microscopy. Proc Natl Acad Sci U S A 99: 2014-2019.

6. Zucker RM, Price OT (1999) Practical confocal microscopy and the evaluation of system performance. Methods 18: 447-458.

7. Gelman L, Rietdorf J (2010) Routine Assessment of Fluorescence Microscope Performance. Imaging & Microscopy: Wiley-VCH Verlag GmbH & Co. KGaA.

http://www.imaging-git.com/science/light-microscopy/routine-assessment-fluorescence-microscope-performance

8. Cole RW, Jinadasa T, Brown CM (2011) Measuring and interpreting point spread functions to determine confocal microscope resolution and ensure quality control. Nature protocols 6: 1929-1941.

9. Adler J, Pagakis SN (2003) Reducing image distortions due to temperature-related microscope stage drift. Journal of microscopy 210: 131-137.
